# Rising ecosystem water demand exacerbates the lengthening of tropical dry seasons

**DOI:** 10.1038/s41467-022-31826-y

**Published:** 2022-07-14

**Authors:** Hao Xu, Xu Lian, Ingrid J. Slette, Hui Yang, Yuan Zhang, Anping Chen, Shilong Piao

**Affiliations:** 1 Sino-French Institute for Earth System Science, College of Urban and Environmental Sciences, Peking University, Beijing, 100871 China; 2 Department of Earth and Environmental Engineering, Columbia University, New York, NY 10027 USA; 3 Department of Biology, Colorado State University, Fort Collins, CO 80523 USA; 4 Graduate Degree Program in Ecology, Colorado State University, Fort Collins, CO 80523 USA; 5 Long Term Ecological Research Network Office, National Center for Ecological Analysis and Synthesis, University of California Santa Barbara, Santa Barbara, CA 93101 USA; 6 Department of Biogeochemical Integration, Max Planck Institute for Biogeochemistry, 07745 Jena, Germany; 7 Laboratoire des Sciences du Climat et de l’Environnement, CEA CNRS UVSQ, Gif-sur-Yvette, 91191 France; 8 State Key Laboratory of Tibetan Plateau Earth System, Resources and Environment, Institute of Tibetan Plateau Research, Chinese Academy of Sciences, Beijing, 100085 China

**Keywords:** Hydrology, Tropical ecology

## Abstract

Precipitation-based assessments show a lengthening of tropical dry seasons under climate change, without considering simultaneous changes in ecosystem water demand. Here, we compare changes in tropical dry season length and timing when dry season is defined as the period when precipitation is less than: its climatological average, potential evapotranspiration, or actual evapotranspiration. While all definitions show more widespread tropical drying than wetting for 1983-2016, we find the largest fraction (48.7%) of tropical land probably experiencing longer dry seasons when dry season is defined as the period when precipitation cannot meet the need of actual evapotranspiration. Southern Amazonia (due to delayed end) and central Africa (due to earlier onset and delayed end) are hotspots of dry season lengthening, with greater certainty when accounting for water demand changes. Therefore, it is necessary to account for changing water demand when characterizing changes in tropical dry periods and ecosystem water deficits.

## Abstract (web summary)

Changing precipitation pattern has been suggested to expand tropical dry seasons. Here, the authors show that this lengthening can be even more severe when accounting for the simultaneous rise of ecosystem water demand in a warmer climate.

## Introduction

Tropical ecosystems, especially rainforests, serve a pivotal role as a natural buffer against global climate change by storing one-half of Earth’s carbon (C) and capturing 1.6 ± 0.5 Pg of C per year^1–3^. The vegetation dynamics and ecosystem functions of tropical systems are sensitive to seasonal rainfall^1,4–6^, with a distinct temporal transition between the dry and wet seasons. As the dry season progresses, declining soil moisture levels caused by precipitation deficit and strong evaporative water loss could shift tropical ecosystems from radiation-limited to moisture-limited^7^ and suppress photosynthesis^7,8^. Hence, in a warmer climate, longer and more intense dry seasons, and the associated enhanced risk of short-term droughts^9^ and fires^10,11^, could reduce tropical ecosystem productivity^12^. Reduced primary productivity and elevated forest mortality due to extended dry seasons could exacerbate forest fragmentation and savannization, which are already of particular concern for tropical rainforests^6,13^, and could exacerbate global warming via biogeochemical and biophysical feedbacks.

The tropical “dry season” is often defined as the period when precipitation (*P*) is persistently lower than the multi-year average precipitation ($\bar{P}$)^10,14–16^. Using this definition, previous studies have consistently suggested a lengthening of dry seasons over the tropics, for example, by ~6.5 d decade^−1^ in southern Amazonia^10^ and by 6.4–10.4 d decade^−1^ in the Congo Basin^16^. However, the long-term dynamics and seasonality of surface water availability are highly complex^17,18^. Land surface dryness depends not only on the supply of precipitation, governed primarily by large-scale atmospheric circulation patterns^19,20^, but also on the rate at which the atmosphere is recycling moisture from the land, which can be measured as potential evapotranspiration (*Ep*) or actual evapotranspiration (*E*)^17,21^. As the global climate changes, the increasing evaporative demand of the warmer atmosphere (i.e., increasing *Ep*) may drive faster soil moisture depletion and cause land surface drying^22,23^. On the other hand, the actual evaporative water loss (*E*) does not necessarily follow the increasing atmospheric demand^24–26^, since it is highly responsive to alterations of ecosystem biophysical properties such as soil moisture, vegetation cover, and stomatal conductance^27,28^.

Recent studies have increasingly accounted for the balance between water supply (*P*) and demand (e.g., *Ep* or *E*) in analyzing the climatology of dry seasons^4,7,12^. However, this balance has rarely been considered when assessing temporal changes in dry seasons. These three metrics ($\bar{P}$, *Ep* and *E*) consider different land- or near-surface processes and emphasize different aspects of water balance^29^. Hence, changes in dry season length, timing, and intensity are likely to vary spatially and temporally, depending on which definition of “dry season” is used. Inconsistent definitions can limit synthesis and inhibit understanding of the impacts of common ecological phenomena^30^. Thus, understanding how the extent of dry season lengthening varies among definitions is necessary for more accurate assessments of the spatiotemporal variations in tropical seasonal water deficit, and for taking effective management measures to mitigate water shortages and associated ecosystem impacts in seasonally dry regions.

In this study, we characterized variations in dry season length (DSL), dry season arrival (DSA) date, and dry season end (DSE) date, as well as the severity of water deficit (WD) over the global tropics (23.5°S-23.5°N) during the period of 1983–2016, among different definitions of the dry season, using multiple combinations of observational and reanalysis datasets (see Methods). In particular, we considered three widely used definitions of “dry season”: (i) *P* < *Ep*, a comparison of water supply vs. atmospheric water demand, (ii) *P* < *E*, a comparison of water supply vs. actual ecosystem water consumption, and (iii) *P* < $\bar{P,}$ a comparison of water supply vs. the long-term mean water supply. Our objective was to understand how the length and timing of the dry season have been changing over the tropics and whether definitions of the dry season that account for atmospheric water demand or actual ecosystem water loss indicate similar or different changes in the length and timing of the tropical dry season, compared to definitions based merely on precipitation.

## Results

### Mean dry season length and timing over tropics

**Fig. 1:**
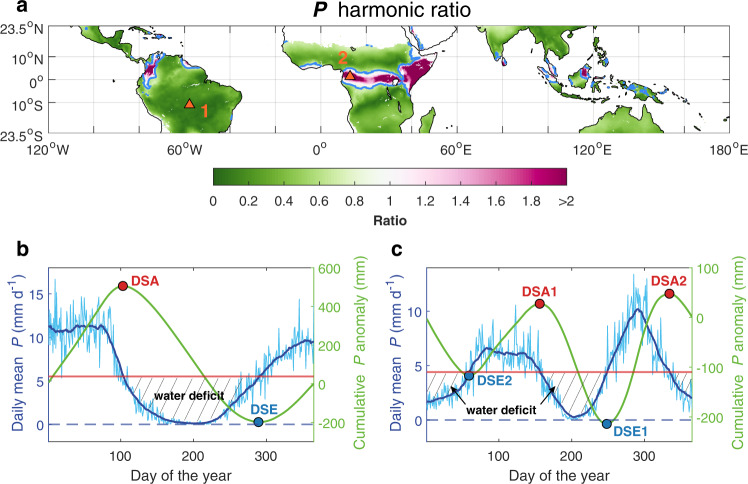
Precipitation seasonality and dry season defined as *P* < $\bar{P}$. **a** The mean ratio of *P* amplitudes of the harmonics at frequencies of two and one cycle per year via Fourier Analysis of whole daily time series (1983–2016) for each grid box based on the eight precipitation datasets. The blue line marks the boundaries with the ratio of 0.75, inside which grid boxes have two wet seasons and two dry seasons per year. **b**, **c** Daily mean rainfall (light blue) for each day of the year, smoothed using a 30-day running window (blue), the multi-year average daily mean precipitation (red horizontal line), and cumulative *P* anomaly value (green) for the grid box centered at 11.125°S, 57.875°W (left, point 1 in **a**) and 1.375°N, 12.875°E (right, point 2 in **a**) according to the daily CHIRPS precipitation dataset for the period 1983–2016 (see Eq. 3). Red dots mark the arrival of dry seasons (DSA), while blue dots mark the end of dry seasons (DSE). The solid black shaded area represents the water deficit, calculated as the cumulative difference between *P* and $\bar{P}$ during the dry season. The two longest seasons are assumed to be the two seasons of interest for the biannual region (**c**). Similar patterns and definitions for *P* < *Ep* and *P* < *E* are shown in Supplementary Figs. 2, 3.

**Fig. 2:**
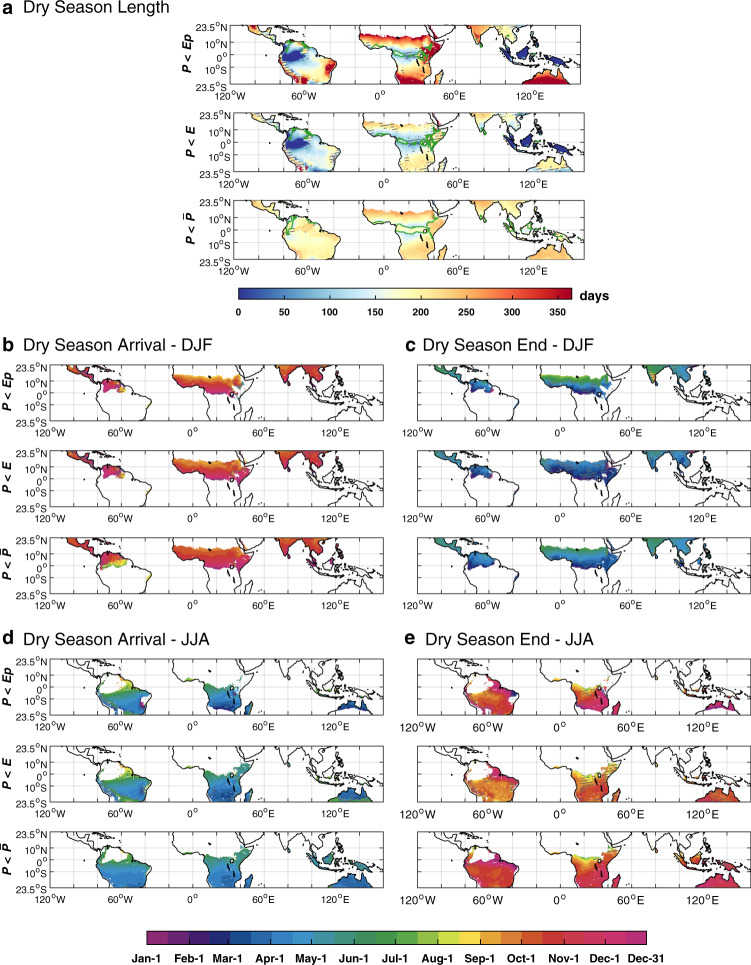
Spatial patterns of tropical dry season length and timing under three definitions. The dry season timing (arrival date and end date) is shown for northern tropics (**b**, **c**) and for southern tropics (**d**, **e**), respectively. In **a** green lines indicate the boundary of bimodal precipitation regimes inside which grid boxes have two dry seasons per year, typically with one in boreal winter (December–February, **b**, **c**) and the other in boreal summer (June–August, **d**, **e**). The hatched area in **a**–**e** indicates that the standard deviation of DSL is longer than 30 days (in **a** and **b**) or the standard deviation of DSA or DSE is longer than 15 days (in **d** and **e**).

Given the complicated seasonality of dry-wet transition over different tropical lands, we first applied a harmonic analysis to precipitation to determine if one or two dry seasons are experienced per year for each grid point (see Methods). We found that the majority (87.4%) of tropical lands have one distinct dry season per year (i.e., a ratio of amplitudes of harmonics < 0.75, see Methods, Fig. 1a), though the timing differs between the northern and southern tropics (Fig. 2b, c). December, January, and February were the driest months in the northern tropics, whereas June, July, and August were the driest months in the southern tropics (Supplementary Fig. 1a–d, Fig. 2). A small proportion of the tropics might experience two dry seasons per year (ratio > 0.75; Fig. 1a), predominantly located near the equator such as the Congo Basin, East Africa, the southern coastal region of West Africa, the northwestern Amazon Basin and the southeastern Asia. Peak precipitation in these bimodal regions usually occurs during the transitional periods March–May and September–November (Fig. 1c), resulting in two main dry seasons during December–February and June–August (Fig. 2). This pattern was mainly shaped by the seasonal progression of the Inter-Tropical Convergence Zone^14^ and tropical monsoon systems^15,31,32^. Analyses using *P* < *Ep* and *P* < *E* produced similar results (Supplementary Fig. 2), indicating that all three definitions were generally sensitive to seasonal transitions between dry and wet periods, which was a premise for comparing changes in dry season length and timing among different definitions.

DSL varied among the three different definitions (see Methods, Fig. 2a). DSL defined as *P* < *Ep* or *P* < *E* ranged from 0 (i.e., no “dry season”) in Amazonia and southeastern Asia rainforests, to longer than 200 days (i.e., “dry season” lasting most of the year) in areas with very low precipitation such as sub-Saharan Africa, the Arabian Peninsula and Australia (Fig. 2a), generally following a latitudinal gradient similar to that of precipitation decrease from the tropical lows to subtropical highs. The longer dry season at higher latitudes was a result of both earlier DSA and delayed DSE (Fig. 2b, c). By contrast, analysis using *P* < $\bar{P}$ showed relatively homogeneous spatial patterns of DSL, with over 86% of tropical areas falling within the range of 150–240 days (Fig. 2a). In particular, for humid regions, such as rainforests, where the other two definitions indicated a short or nonexistent dry season, *P* < $\bar{P}$ indicated a dry season lasting >150 days, comparable to regions with relatively dry climates. These patterns were consistent for areas that experience both one and two dry seasons per year (Fig. 2d, e). Further analyses revealed that the discrepancy of DSL among different precipitation datasets is much smaller than that among definitions (Fig. 2). DSL defined by *P* < $\bar{P}$ was the least sensitive to precipitation inputs, as the $\bar{P}$ self-adjusted for different precipitation datasets.

**Fig. 3:**
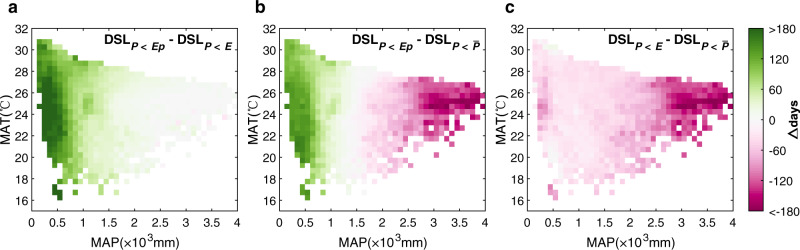
Inter-metric differences in dry season length by mean annual climate. **a**–**c** The differences in dry season length (DSL) were calculated among the three definitions (*P* < *Ep*, *P* < *E*, and *P* < $\bar{P}$), and averaged within each mean annual temperature (MAT) and mean annual precipitation (MAP) interval from ERA-5. Pixel number for each MAT and MAP interval in **a**–**c** is shown in Supplementary Fig. 5.

We found that the inter-metric DSL difference varied according to regional mean annual precipitation (MAP), due to the relationship between *Ep* and *E* (Supplementary Fig. 6). Under arid conditions (that is, when *P* is much smaller than *Ep*), actual water consumption, *E*, is mainly limited by the available supply of surface water, which is much smaller than the *Ep* and converges toward the multi-year average rainfall $\bar{P}$. Therefore, DSL was similar when defined as either *P* < $\bar{P}$ or *P* < *E* but was longer when defined as *P* < *Ep* (Fig. 3). This difference increased from 30–40 days with MAP ~1000 mm yr^−1^ to 170–190 days with MAP ~200 mm yr^−1^. Alternatively, under humid conditions (i.e., when *P* is much greater than *Ep*), *E* is mainly limited by atmospheric water demand and converges toward *Ep*. DSL was similar when defined as either *P* < *E* or *P* < *Ep* but was longer when defined as *P* < $\bar{P}$ (Fig. 3). This difference increased from 60 to 120 days in areas with MAP of 2000–2500 mm yr^−1^, to >120 days with MAP > 2500 mm yr^−1^. Meanwhile, ~39.2% of the tropical grids, with MAP between 1000 and 1500 mm yr^−1^, showed little difference in DSL between any two definitions.

### Extent of dry season lengthening and water deficit increase

**Fig. 4:**
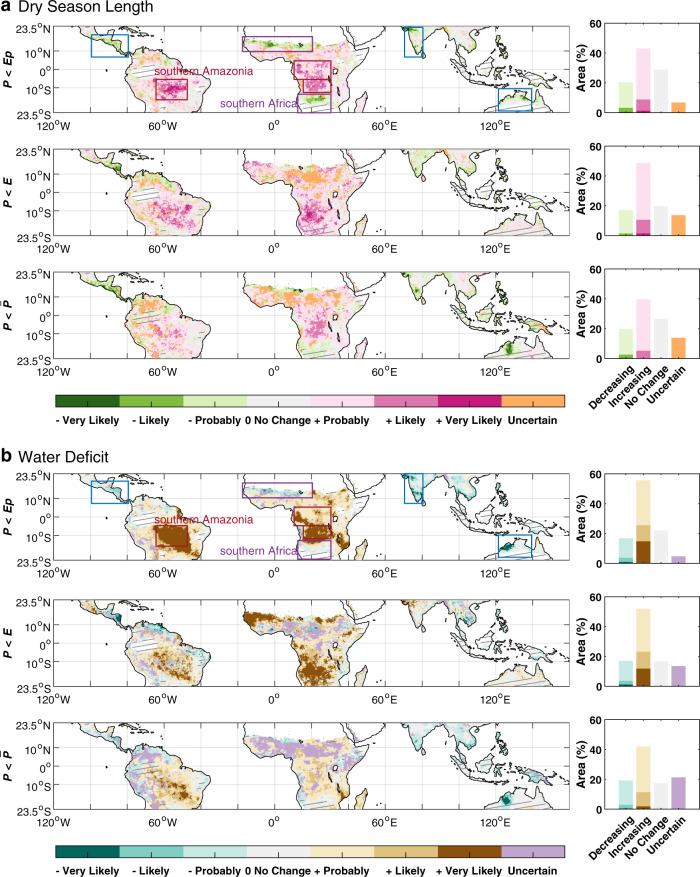
Consistency of trends in dry season length and water deficit for each definition, from eight datasets (1983-2016). The consistency of trends in dry season length (**a**) and water deficit (**b**) under each of the three definitions of “dry season” was assessed as the variation among the eight precipitation datasets. “Very likely”, ‘Likely” and “Probably” indicate that the sign of the trend was the same and significant in 6–8, 4–5, and 1–3 precipitation datasets, respectively, while the other datasets showed no significant change. “Uncertain” indicates conflicting trends among datasets, with some showing a significant increase and some showing a significant decrease. “No Change” indicates that all eight datasets showed no significant change. The histograms in the right column of **a** and **b** show the percent area with consistent increase or decrease trends. Arid and humid regions (solid gray shaded area) were excluded when calculating the percent area, since there was no climatologically wet or dry season, thus no trends calculated under definitions of *P* < *Ep* or *P* < *E*. This analysis combined both dry seasons for regions with two dry seasons (individual trends for each distinctive season are shown in Supplementary Fig. 9). Consistency of trends in dry season length and water deficit for the other two *Ep* products (MERRA-2 and GLDAS-v2.0) based on *P* < *Ep* definition were shown in Supplementary Fig. 8.

We next assessed long-term trends in dry season length and timing (DSL, DSA, and DSE) as well as the water deficit (WD, defined as the cumulative difference between *P* vs. *Ep*, *E*, or $\bar{P}$) during the dry season using each of the three definitions. All the three definitions indicated more widespread drying than wetting over the tropics (Fig. 4). However, the extent of land area experiencing a drying trend varied considerably among these different definitions. The overall fraction of tropical land area with lengthening dry seasons (at least one significant drying trend, and no significant wetting trend, among different datasets) was largest when dry season was defined as *P* < *E* (48.7%), followed by *P* < *Ep* (43.1%), and was smallest for *P* < $\bar{P}$ (33.7%). Accordingly, a smaller fraction of regions was probably experiencing shorter dry seasons (thus longer wet seasons), accounting for ~20.4%, 17.1%, and 19.8% of tropical lands when dry season was defined as *P* < *Ep*, *P* < *E,* and *P* < $\bar{P}$, respectively (Fig. 4a). We also found that the extension of the dry season was accompanied by an increase in the cumulative WD during the dry season (Fig. 4b). The percent of tropical land area experiencing increasing dry season water deficit (~55.7%, 51.7%, and 38.0% for *P* < *Ep*, *P* < *E*, and *P* < $\bar{P}$, respectively) was larger than that experiencing decreasing water deficit (~16.8%, 17.5%, 19.2% for *P* < *Ep*, *P* < *E,* and *P* < $\bar{P}$, respectively). Analyses using other two independent *Ep* products detected similar fraction of lengthening dry seasons (MERRA-2: 41.5%; GLDASv2.0: 42.9%) and increasing water deficit (MERRA-2: 49.9%; GLDASv2.0: 52.0%), indicating a robust drying trend among different *Ep* datasets (Supplementary Fig. 8).

The three dry season definitions consistently identified some regions with robust dry season lengthening (more than four out of eight datasets agree), including southern Amazonia and central Africa (red rectangles in Fig. 4). The increased DSL in southern Amazonia (4.81–10.84, 3.65–11.96, and 5.95–10.53 d decade^−1^ when defined as *P* < *Ep*, *P* < *E*, and *P* < $\bar{P}$, respectively, Supplementary Table 2) was mainly attributed to a delayed DSE (Supplementary Fig. 7). In the Congo Basin and southern central Africa, the length of the June–August dry season increased by 7.62–11.61, 6.62–11.65, and 8.19–11.93 d decade^−1^ when defined as *P* < *Ep*, *P* < *E*, and *P* < $\bar{P}$, respectively (Fig. 4, Supplementary Table 2). This dry season extension in central Africa was due to both a delayed DSE and an earlier DSA (Supplementary Fig. 7). For both southern Amazonia and central Africa, the dry season lengthening and associated increased water deficit were most robust under the definition of *P* < *Ep*, followed by *P* < *E* (Fig. 4, Supplementary Table 2).

**Fig. 5:**
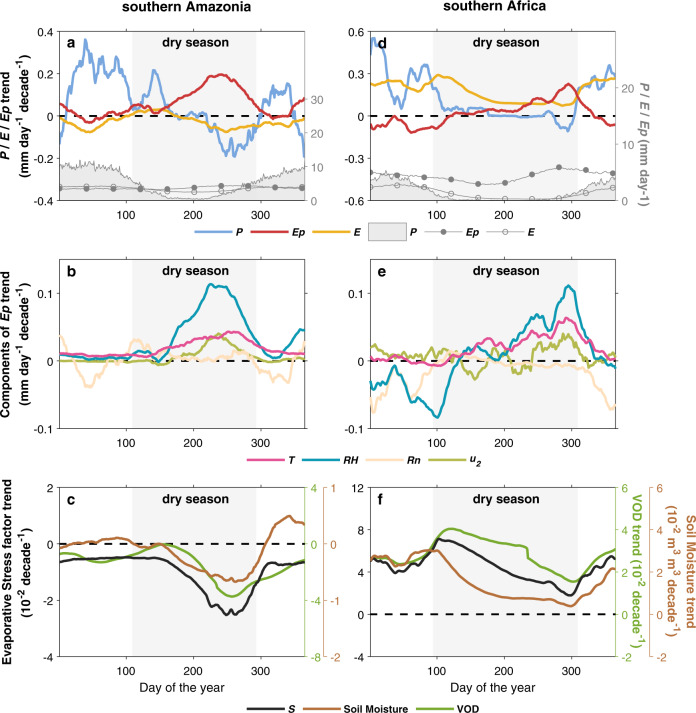
Seasonal trajectories of *P*, *Ep*, *E* changes and the drivers in southern Amazonia and southern Africa. For each day of the year, the linear trends were calculated with the zonally averaged and 30-day smoothed daily values over southern Amazonia (48–65°W, 5−16°S, **a**–**c**) and southern Africa (12-30°E, 13-23.5°S, **d**–**f**), over the 1983-2016 period. **a**, **d** These show the daily means and overall trends of *P, Ep*, and *E*. The gray area (in **a** and **d**) indicates the dry season based on *P* < $\bar{P}$. **b**, **e** They show the individual contributions of *T*, *RH*, *u*_*2*_, and *Rn* to the overall trend of *Ep*. **c**, **f** These show the changes of parameters (*S*, VOD, *S*oil Moisture) representing the constraints of soil moisture and vegetation water content on *E*. Datasets included in this analysis are: *P* from MSWEP v2.8, *Ep*, *T*, *RH*, *u*_*2*_, and *Rn* from ERA-5*. E*, Stress factor and Soil Moisture from GLEAM v3.3a, Vegetation Optical Depth from VODCA Ku-band (available only for 1987–2016).

In southern Africa and Sahel, different definitions suggested different trends (purple rectangles in Fig. 4). In southwestern Africa, for example, all eight datasets indicated dry season lengthening (5.30–16.51 d decade^−1^) due to delayed DSE, when defined as *P* < *E* (Fig. 4, Supplementary Table 2, Supplementary Fig. 7). By contrast, we found a shortening of DSL (−5.66 to −9.24 d decades^−1^) when dry season was defined as *P* < *Ep*, and no robust change in DSL when it was defined as *P* < $\bar{P}$ (Fig. 4, Supplementary Table 2). This contradictory result was primarily due to the inconsistent trends of ecosystem *E* (significant increases by ~0.14 mm d^−1^ decade^−1^) and *Ep* (decreases by ~0.17 mm d^−1^ decade^−1^), while *P* showed little changes (Fig. 5).

Compared to the widespread drying trend, the wetting trend was mainly scattered in central America, India, and northwestern Australia (blue rectangle in Fig. 4). The shortening of the dry season (~5–15 d decade^−1^) over these regions was generally caused by a delayed DSA as well as an advanced DSE (Supplementary Fig. 7). It is worth noting that a large proportion of tropical land area (35.8%, 33.4%, 40.5%, respectively) was not decisively experiencing a longer or shorter dry seasons (labeled as “No change” or “Uncertain” in Fig. 4), indicating that not everywhere was experiencing a significant trend in DSL. This could also be due to inter-data discrepancy of precipitation changes, primarily due to the paucity of rainfall observation gauges or biases of the models for reanalyzing (see Methods).

Our observational finding of the exacerbated lengthening of tropical dry seasons by rising water demand was also supported by climate model simulations from the Coupled Model Intercomparison Project Phase 6 (CMIP6) (see Methods), which in general showed more widespread and robust drying trends based on *P* < *Ep* than the other definitions (Supplementary Fig. 11). However, significant observation-model discrepancies also existed for the extent and locales of tropical DSL changes. For instance, model-derived areas experiencing lengthening dry seasons were the smallest when inferred from *P* < *E* (13.4%, compared to 48.7% with observational data under the same definition), while they were smallest when inferred from *P* < $\bar{P}$ for observation-derived results (Fig. 4). Furthermore, the robust dry season lengthening detected over southern Africa and Sahel based on observed *P* < *E* is not present in CMIP6 model results (Fig. 4, Supplementary Fig. 11). This model-observation discrepancy is likely attributable to plant physiological responses to rising atmospheric CO_2_. Plants partially close their leaf stomata under rising atmospheric CO_2_, which reduces plant water loss and reduces the increase of evapotranspiration^33–35^. Fully coupled Earth system models explicitly consider the hydrological consequences of plant physiological responses to rising CO_2_. For example, modeling studies identify this CO_2_-related mechanism as a critical factor of tropical *E* changes and seasonality of tropical hydrology^33,34^. However, this physiological factor is currently absent in calculating terrestrial *E* from observed climate and surface data, based on empirically- or physically-models, which may lead to an overestimation of inferred drying trends in observational analyses. On the other hand, CO_2_ fertilization may enhance leaf area, which could cancel the water-saving from increased water use efficiency^36,37^. Although the leaf- to ecosystem-level increases of water-use efficiency and leaf area under higher CO_2_ have been well-studied with laboratory and field experiments^38^, it still remains largely unknown how these processes may affect surface water availability at broader spatial scales^39^.

### Decoupled trends of dry-season *Ep* and *E*

To better illustrate how water demand or actual water loss affects the length of the dry season, we further analyzed the potential drivers of changes in dry-season *Ep* and *E* in regions with robust drying trends (See Methods, Fig. 5). During the dry season, precipitation decline was often accompanied by rising atmospheric water demand, primarily due to humidity decrease especially in the late dry season (Fig. 5). For instance, in southern Amazonia, the prolonged dry season was initialized by the precipitation decline during the late dry season (Fig. 5a), followed by a prominent increase in *Ep* mainly (>50%) due to decreasing air humidity from insufficient water supply of rainfall. Rising temperature and increasing wind speed also partially exacerbated the atmospheric drying, while the effect of radiation changes was negligible (Fig. 5b). The rising atmospheric water demand that driven by decreasing air humidity and rising temperature were also identified in the other two *Ep* datasets (Supplementary Fig. 12). Hence, compared with the *P*-only trend in DSL, the enhanced atmospheric water demand exacerbated the drying trend (Fig. 4). Towards the end of dry season when rainfall started to increase, *Ep* decreased simultaneously (Fig. 5a–d).

However, the thermodynamically-driven increase in water demand (i.e., *Ep*) does not translate into similar growth in actual water loss (i.e., *E*) during the dry season, which additionally depends on available water for evaporation. The persistent rainfall deficit and warming-induced depletion of soil moisture caused an ongoing decline in soil moisture (Fig. 5c). Less available soil moisture and higher atmospheric water demand typically drove plants to partially close their stomata to reduce water loss and avoid critical xylem embolism, evidenced by a decrease in vegetation water content (measured by Vegetation Optical Depth in Fig. 5c). Thus, the evaporative stress of both soil evaporation and plant transpiration was intensified (i.e., decreasing stress factor), leading to a slower increase of actual *E* than potential *E* (*Ep*). This mechanism underlies the more moderate drying trend based on *P* < *E* (compared with that based on *P* < *Ep*) in regions such as southern Amazonia and Congo Basin. In southern Amazonia, *E* even showed an opposite decreasing trend in dry season with strong constraints by limited water supply (Fig. 5a).

Still, anomalous *E* increase during the dry season has been observed in regions such as southern Africa, due to the buffering effect of surface water storage. We found that precipitation has increased during the rainy and transitional seasons (Fig. 5d), resulting in more water storage in soil and plants, which can persist into the following dry season and supplement water use under stressed conditions (Fig. 5f). Although there was no extra water supply from precipitation during the dry season, actual *E* increased continuously from the rainy season to the dry season (Fig. 5c) and caused a drying trend based on *P* < *E*. This result indicates that to some extent water stress for vegetation and evaporation during the dry season is also influenced by water supply and storage from the preceding wet season.

## Discussion

The changes in dry season, including its length and timing, has been an increasingly important issue for tropical ecosystem dynamics under climate change. We showed that the extent of dry season lengthening over the global tropics varied depending on the definition of “dry season”. The most commonly employed definition of dry season as *P* < $\bar{P}$, which has extensively used in studies assessing tropical dry season changes and associated ecosystem responses^10,14–16^, only requires precipitation as input and thus measures seasonal deficits of atmospheric water supply. Dry seasons identified by this metric were reported consistent with local agricultural definitions^14^ and can serve as an important parameter in agricultural practices^40^. This metric divides the whole year into the relatively dry and wet periods, roughly half by half (Fig. 2). With this metric, research has identified robust dry season lengthening for some tropical regions^10,14,16^. In particular, a strong lengthening of the dry season by precipitation observations has been found in the southern Amazonia^10,24^ and the Congo Basin^16^, which is usually interpreted by the changes of large-scale atmospheric circulation^41^, as well as feedbacks of regional deforestation on precipitation^42–44^.

The extension of the dry season is even more robust and widespread when it is defined as *P* < *Ep* or *P* < *E* (vs. *P* < $\bar{P}$) (Fig. 4). These two metrics define dry and wet conditions according to a balance between water supply vs. demand or evaporative water loss at the surface (respectively)^7,12^. In a warming and drying climate, decreased humidity and increased temperature have significantly contributed to the increase in atmospheric water demand^21–23^ in some regions, including the southern Amazonia and central Africa (Fig. 4), thus exacerbating the drying trend. In addition, increasing *E* due to alleviated vegetation water stress^28^ has a greater impact than precipitation changes, and thus causes a further drying trend (based on the definition of *P* < *E*) in regions such as southern Africa (Figs. 4, 5). Hence, the numerous previous studies that are based only on *P* < $\bar{P}$ and lack consideration of the increasing water demand of the atmosphere or ecosystems in a warmer climate may underestimate the prolongation of the dry season and the exacerbation of the ecosystem water deficit.

Further, studies which only consider precipitation change may not fully capture ecosystem responses to prolonged dry seasons, as ecosystem dynamics can be strongly affected by the water availability. For example, in the tropical wet-dry transition season, extended periods of high atmosphere water demand (measured by the vapor pressure deficit) have been acknowledged as a primary driver of large-scale tree mortality and wildfire in forest ecosystems^11,45–47^. Previous work has also indicated that, during the dry season, photosynthesis and vegetation productivity in tropical rainforests are mainly constrained by water demand^7,48,49^. Thus, because primary production in the tropics plays an essential role in global C cycling and the size of the land C sink, global C dynamics have likely been altered by changes in tropical DSL and dry season water demand. Mounting evidence shows that the *P* < *Ep* metric has been observed to decrease (indicative of surface drying) under current warming trend and is expected to continue into the future^21,50^. Therefore, with accounting for warming-driven increase in water demand, the *P* < *Ep* definition is likely more suitable for studying potential responses of ecosystem productivity to demand-driven dry season changes, particularly on the increasing risk of heat, drought, and wildfire disturbances in a warmer and drier climate.

Considering that the actual amount of ecosystem water consumption, *E*, involves effects of complex land surface processes on the hydrological cycle, dry season definition based on *P* < *E* could capture seasonal changes in surface water resources more accurately. Due to the soil moisture and vegetation phenological constraints, *E* trend can be decoupled from *Ep* trend on seasonal to longer time scales^24–26,51–53^. *Ep* is calculated by assuming the land surface is not water-limited, hence it cannot capture *E* changes in relatively dry periods or over extremely dry regions^24,52^. For example, in southern Amazonia, soil moisture and vegetation phenology constraints have strongly limited the evaporation water loss (Fig. 5). Furthermore, human land-use activities could have a more substantial impact on *E* than on *P* or *Ep*. For example, widespread forest clearing over the tropics may reduce precipitation^41,54–56^, but the actual water limitation is eased since the degraded ecosystems demand less water for growth^51,57^.

In summary, our study reveals that the extent of dry season lengthening over the global tropics varies depending on the definition of “dry season”. Considering changes in water demand or actual water loss exacerbates tropical dry season lengthening. Climate change not only alters the precipitation regimes of the global tropics, it also changes the demand side of the ecosystem water cycling that has strong impact on tropical vegetation dynamics and ecosystem carbon cycling. Therefore, in order to more fully capture ecosystem response, we recommend that future studies account for changing water demand when characterizing changes in seasonal dry periods and ecosystem water deficits in an increasingly warmer and drier climate.

## Methods

### Climate and land cover data

Our study of tropical dry season dynamics required climatic variables with high temporal resolution (i.e., daily) and full coverage of tropic regions. To reduce uncertainties associated with the choice of precipitation (*P*) and evapotranspiration (*Ep* or *E*) datasets, we used an ensemble of eight precipitation products, three reanalysis-based products for *Ep,* and one satellite-based land *E* product. These precipitation datasets were derived four gauge-based or satellite observation (CHIRPS^58^, GPCC^59^, CPC-U^60^ and PERSIANN-CDR^61^), three reanalyses (ERA-5^62^, MERRA-2^63^, and PGF^64^) and a multi-source weighted ensemble product (MSWEP v2.8^65^). The potential evapotranspiration (*Ep*) was calculated using the FAO Penman–Monteith equation^66^ (Eqs. (1, 2)), which requires meteorological inputs of wind speed, net radiation, air temperature, specific humidity, and surface pressure. We derived these meteorological variables from the three reanalysis products (ERA-5, MERRA-2, and GLDAS-2.0^67^). Since PGF reanalysis lacked upward short- and long-wave radiation output and thus net radiation, we used available meteorological outputs from GLDAS-2.0 instead, which was forced entirely with the PGF input data.

1 $${Ep}=\frac{0.408\cdot \triangle \cdot \left({R}_{n}-G\right)+\gamma \cdot \frac{900}{T+273}\cdot {u}_{2}\cdot \left({e}_{s}-{e}_{a}\right)}{\triangle +{{{{{\rm{\gamma }}}}}}\cdot \left(1+0.34\cdot {u}_{2}\right)}$$

2 $${VPD}={e}_{s}-{e}_{a}=0.6108\cdot {e}^{\frac{17.27\cdot T}{T+237.3}}\cdot \left(1-\frac{{RH}}{100}\right)$$

Where *Ep* is the potential evapotranspiration (mm day^−1^). *R*_*n*_ is net radiation at the surface (MJ m^−2^ day^−1^), *T* is mean daily air temperature at 2 m height (°C), ${u}_{2}$ is wind speed at 2 m height (m s^−1^), ($\,{e}_{s}-{e}_{a}$) is the vapor pressure deficit of the air (kPa), ${RH}$ is the relative air humidity near surface (%), *∆* is the slope of the saturation vapor pressure-temperature relationship (kPa °C^−1^), *γ* is the psychrometric constant (kPa °C^−1^), *G* is the soil heat flux (MJ m^−2^ day^−1^, is often ignored for daily time steps *G* ≈ 0).

We derived the daily evapotranspiration data from the Global Land Evaporation Amsterdam Model (GLEAM v3.3a^68^), which is a set of algorithms dedicated to developing terrestrial evaporation and root-zone soil moisture data. GLEAM fully assimilated the satellite-based soil moisture estimates from ESA CCI, microwave L-band vegetation optical depth (VOD), reanalysis-based temperature and radiation, and multi-source precipitation forcings. The direct assimilation of observed soil moisture allowed us to detect true soil moisture dynamic and its impacts on evapotranspiration. Besides, the incorporation of VOD, which is closely linked to vegetation water content^69,70^, allowed us to detect the effect of water stress, heat stress, and vegetation phenological constraints on evaporation. Other observation-driven ET products from remote-sensing physical estimation and flux-tower are not included due to their low temporal resolution (i.e., monthly)^71^ or short duration^72,73^. ET outputs of reanalysis products are not considered in our analysis, because the assimilation systems lack explicit representation of inter-annual variability of vegetation activities and thus may not fully capture hydrological response to vegetation changes^62,63,67^.

We used land cover maps for the year 2001 from the Moderate-Resolution Imaging Spectroradiometer (MODIS, MCD12C1 C5^74^) based on the IGBP classification scheme to exclude water-dominated and sparely-vegetated pixels (like Sahara, Arabian Peninsula). All climate and land cover datasets mentioned above were remapped to a common 0.25° × 0.25° grid and unified to daily resolution. The main characteristics of the datasets mentioned above are summarized in Supplementary Table 1.

### Outputs of CMIP6 simulations

To understand how modeled dry season changes compare with observed changes, we analyzed outputs from the “historical” (1983-2014) runs of 34 coupled models participating in the 6th Coupled Model Inter-comparison Project^75^ (CMIP6, Supplementary Table 3). We used these models because they offered daily outputs of all climatic variables needed for our analysis, including precipitation, latent heat (convert to *E*), and multiple meteorological variables for *Ep* (air temperature, surface specific humidity, wind speed, and net radiation). All outputs were remapped to a common 1.0° × 1.0° grid and unified to daily resolution.

### Defining dry season length and timing

For each grid cell and each dry season definition (*P* < *Ep*, *P* < *E* and *P* < $\bar{P}$), we conducted a harmonic analysis to define the number of dry and wet seasons experienced per year, through a Fourier transform of the entire daily time series^15,16,76^. We calculated the ratio of harmonic amplitudes at frequencies of one and two cycles per year to determine seasonality (Fig. 1a, Supplementary Fig. 2). A ratio greater than 0.75 indicates that the harmonic of two cycles per year (i.e., two dry and wet seasons, Fig. 1c) may fit the time series better, otherwise (ratio < 0.75) there is more likely a single dry/wet season (Fig. 1b).

The three definitions of “dry season” that we assessed were: (i) the period when daily precipitation (*P*) is persistently less than daily potential evapotranspiration (*Ep*), i.e., *P* < *Ep*, (ii) the period when daily precipitation is persistently less than daily actual evaporation (*E*) i.e., *P* < *Ep*, and (iii) the period when daily precipitation is persistently less than the multi-year average daily mean precipitation $\left(\bar{P}\right.$), i.e., *P* < $\bar{P}$. Other definitions of “dry season” (e.g., based on a specific rainfall threshold^77,78^) have been used in previous research. We chose these three definitions because they can be applied across the entire tropical land area (i.e., they are not locally determined by metrics such as a specific local rainfall threshold value). The dry season should be continuous, not the total number of intermittent dry days. We ensured the continuity for the definition with two rules: (1) regions with bimodal rainfall regime were identified through previous harmonic analysis and discussed separately, for which each single dry season should be continuous, (2) we adjusted the widely-used *P* < $\bar{P}$ dry season algorithm^15,16^ to identify the arrival and end of the dry season for all the three definitions, which can avoid the influence of short-term climate anomalies. First, we calculated the mean *P, Ep,* and *E* for each day (*j*) of the calendar year (*P*_*j*_*, Ep*_*j*_, and *E*_*j*_) and the daily mean rainfall $\bar{P}$ for all datasets for 1983-2016. To reduce the synoptic noise, we smoothed *P, Ep,* and *E* with a 30-day running window. Then, we calculated cumulative *P − Ep, P − E*, and *P* − $\bar{P}$ on day *d*, ranging from 1 Jan to 31 Dec, as:

3 $$A\left(d\right)=\mathop{\sum }\limits_{j=1}^{d}{P}_{j}-{{Ep}}_{j}$$

4 $$B\left(d\right)=\mathop{\sum }\limits_{j=1}^{d}{P}_{j}-{E}_{j}$$

5 $$C\left(d\right)=\mathop{\sum }\limits_{j=1}^{d}{P}_{j}-\bar{P}$$

*A(d), B(d)*, and *C(d)* increase at day *d* when the daily precipitation is above the daily mean rainfall, daily potential evapotranspiration or actual evaporation, and decrease when the daily precipitation is below the corresponding diagnostic criterion. We defined the day of maximum *A(d), B(d),* or *C(d)* as the arrival of the climatological dry season (DSA) and the day of minimum cumulative value as the end of the climatological dry season (DSE). For regions with two or more dry seasons per year, we detected all days of maximum and minimum in the cumulative curve (Fig. 1c), but we used only the four days marking arrivals (*dsa1*, *dsa2*) and ends (*dse1*, *dse2*) of the two longest dry seasons for our analysis, usually a boreal summer (June–August) dry season and a boreal winter (December–February) dry season.

We calculated DSA, DSE, and DSL under each definition for each dataset (Supplementary Fig. 4), and we calculated the mean DSA, DSE, and DSL under each definition in Fig. 2 and Fig. 3. The mean annual precipitation values and the mean annual temperature values in Fig. 3 were derived from the ERA-5 datasets. We examined the uncertainty by calculating the standard deviation among all ensembles of *P*, *Ep,* and *E* under each definition.

To assess temporal changes, we calculated the arrival and end dates individually for each year from 1983 to 2016. We calculated the cumulative *A(d), B(d),* and *C(d)* for each day from DSA − 60 to DSE + 60 for each year instead of the entire calendar year from 1 Jan to 31 Dec, to ensure the correct season was captured. Since the dry season may potentially span multiple calendar years, the dry season arrival and end are not computed for the first and last year of each record. For regions with two dry seasons, the arrival and end dates were determined for the two dry seasons separately. For those regions, we calculated the cumulative function *A(d), B(d)*, and *C(d)* for each day during DSA1 − 45 to DSE1 + 45 (for the first dry season detection) and DSA2 − 45 to DSE2 + 45 (for the second dry season detection). We used a shorter period (45 days, as opposed to 60 days used for regions with one dry season) in order to better capture the characteristics of the two dry seasons. Accordingly, DSL in days can be calculated as the difference between DSE and DSA, or between DSE1 + DSE2 and DSA1 + DSA2 for cases of two dry seasons. We calculated Water Deficit (WD) as the cumulative sum of *P* − $\bar{P}$*, P−Ep*, or *P−E* (dashed area in Fig. 1a), from the dates of DSA to DSE.

### Long-term trend analysis

To assess temporal changes, we calculated annual dry season diagnostics (DSL, WD, DSA, DSE) individually for each year from 1983 to 2016. We estimated the trends of dry season diagnostics and climatic variables from the ordinary least squares linear regression. We defined each trend as the slope of this linear regression, and we determined statistical significance (*P* value) using two-tailed Student’s *t* tests. We used the nonparametric Mann–Kendall trend test to detect whether a significant monotonic increasing or decreasing trend exists, and to provide additional verification for the robustness of the linear regression trend analysis, as it is less sensitive to the beginning and end of the analysis period^16^. In addition, we calculated the time series of DSL, DSA, DSE, WD, and meteorological variables at the regional aggregated level using area-weighted averaging over the southern Amazonia, northern and southern central Africa, and southwestern Africa, to maximize large-scale features while minimizing local-scale variability and noise^16^. We estimated the linear trends at the regional level as at the grid level (Supplementary Table 2).

Considering the inconsistency of trends across precipitation and evapotranspiration datasets, we judged the level of consistency with the following criterion^29^: “very likely” if the sign of the trend was the same and significant (*P* < 0.05) in six to eight precipitation datasets and no significant changes in the others, “likely” if the sign of the trend was supported by four or five precipitation datasets, “probably” if the sign of the trend was supported by one to three, “uncertain” when conflict trends (i.e., both significant increase and decrease trends existed) were found among different precipitation data sources, and “no change” when no significant changes for all of the six datasets were detected. Arid and humid regions (solid gray shaded area in Fig. 4) were excluded when calculating the percent area, since there is no climatology wet or dry season, thus no trends calculated under definitions of *P* < *Ep* or *P* < *E*.

### Driving factors of *Ep* and *E* changes

To further illustrate the thermodynamic mechanism driving higher atmospheric water demand, we disaggregated the individual contributions of four meteorological variables (i.e., *T*, *RH*, *u*_*2*_, and *Rn*) to the *Ep* trends. We derived the contribution of a certain meteorological variable *I* to *Ep* change (*C*_*I*_) as the difference between the *Ep* calculated with all variables changing (i.e., ALL) and that calculated with *I* fixed at its daily climatological values (i.e., *I*_clim_) (Eq. 6). *I* can be air temperature *T*, air humidity *RH*, surface wind speed *u*_*2*_ or surface net radiation *Rn*. We calculated the linear trends of *Ep* and the respective contributions of meteorological variables for the period 1983–2016 (Fig. 5).

6 $${C}_{I}={{Ep}}_{{{{{{\rm{ALL}}}}}}}-{{Ep}}_{{I}_{{{{{{\rm{clim}}}}}}}}$$

As for *E*, GLEAM estimated this flux through reducing *Ep* by an evaporative stress factor (*S; E* = *Ep* × *S + Ei*), based on satellite observations of Vegetation Optical Depth (VOD) and assimilated soil moisture^68^. The latter are calculated using a multi-layer running-water balance. Interception loss (*Ei*) is calculated separately in GLEAM using a Gash analytical model, but its contribution to overall *E* changes was negligible. Hence, we analyzed changes of these parameters (*S*, VOD, Soil Moisture) representing the constraints of soil moisture and vegetation water content on evaporation. Daily VOD was derived from VODCA Ku-band^79^, but only available for the period 1987–2016.

### Data uncertainties

Due to the insufficient and unevenly distributed observation^80^ in the rainfall data over tropics, we integrated daily meteorological station recode (Supplementary Fig. 13), gauge-based, satellite-combined, and reanalysis datasets to study the variations in precipitation and associated dry season change. Our analyses unravel an overall trend of tropic dry season lengthening and identifying some hotspot regions of changes. However, there are some discrepancies in regions like central Africa and Amazon Basin that may have resulted from data uncertainties and the different approaches used to generate homogeneous climate records. Gauge-based and satellite-combined datasets are quite sensitive to the number and density of observations used, but the observational station is sparse in these regions with large number of missing values in daily record (Supplementary Fig. 13). Different interpolation methods were adopted to fill data gaps and produce grid data, which might have generated errors in the rainfall products. For reanalysis, uncertainties are mainly caused by the biases in reanalyzing models, especially in regions with intricacy land surface process, such as Amazon rainforest.

For the *Ep* datasets, we used three independent sets of reanalysis data to verify the changes of atmospheric water demand. Our analyses indicate consistent rising in dry-season water demand, which exacerbated the lengthening of tropical dry seasons, from all datasets in southern Amazonia and southern central Africa. However, only a single dataset was used for *E*, due to the limited data availability at daily intervals, so uncertainty in evapotranspiration estimation has not been fully considered. Actual terrestrial evapotranspiration was modulated not only by surface meteorological conditions and soil moisture but also by the physiology and structures of plants. Changes in nonclimatic factors such as elevated atmospheric CO_2_, nitrogen deposition, and land covers also serve as influential drivers. Uncertainties from those complex processes all contributed to the unclear uncertainty in *E* estimation. Therefore, more efforts should be made to identify and reduce these uncertainties.

## Supplementary information

Supplementary Information

## Data Availability

All observational and reanalysis datasets that we used are publicly available. The daily CHIRPS precipitation datasets are available from the following location: ftp://chg-ftpout.geog.ucsb.edu/pub/org/chg/products/CHIRPS-latest/. The daily satellite-observed TRMM 3B42 precipitation datasets are available at https://disc2.gesdisc.eosdis.nasa.gov/data/TRMM_L3/TRMM_3B42_Daily.7/. The daily CPC-U precipitation data are available at https://psl.noaa.gov/data/gridded/data.cpc.globalprecip.html. The daily GPCC precipitation datasets are available at https://www.dwd.de/EN/ourservices/gpcc/gpcc.html. The daily PERSIANN-CDR precipitation data are available at https://www.ncei.noaa.gov/data/precipitation-persiann/access/. The daily MSWEP v2.8 precipitation data can be obtained from http://www.gloh2o.org/mswep/. The Reanalysis products MERRA-2 are available at https://disc.gsfc.nasa.gov/datasets?project=MERRA-2. The climate variables of ERA-5 reanalysis are available at https://cds.climate.copernicus.eu/cdsapp#!/dataset/reanalysis-era5-single-levels?tab=overview. The PGF reanalysis precipitation product is available at http://hydrology.princeton.edu/data/pgf/v3/0.25deg/daily/. The GLEAM v3.3a datasets are available at https://www.gleam.eu. The land cover product from MODIS (MCD12C1 C5) is available at ftp://ftp.mpic.de/Kaiser/MODIS_land_cover/0.05deg/. The VODCA products are open access at 10.5281/zenodo.2575599.

## Code availability

All computer codes for the process and analysis of the data are available from the corresponding author upon reasonable request.
