# Supplementary material for: Rising ecosystem water demand exacerbates the lengthening of tropical dry seasons
Source: Nat Commun. 2022 Jul 14;13:4093. doi: 10.1038/s41467-022-31826-y (PMC9283447; doi:10.1038/s41467-022-31826-y)
Supplement: Supplementary file 1 — Supplementary Information [file 41467_2022_31826_MOESM1_ESM.docx]

**Supplementary Information for**

**Rising ecosystem water demand exacerbates the lengthening of tropical dry seasons**

Xu et al.

This file includes:

Supplementary Table 1–3, Figures 1–13, and References

**Supplementary Table 1. Precipitation and Evapotranspiration Datasets in this study.** All *P* datasets were interpolated to a common 0.25° grid.

| Datasets | Description | Available Period | Resolution | | Reference |
| --- | --- | --- | --- | --- | --- |
|  |  |  | **spatial** | **temporal** |  |
| *Precipitation* | | | | | |
| PERSIANN-CDR | Precipitation Estimation from Remotely Sensed Information using Artificial Neural Networks | 1983-2018 | 0.25° × 0.25° | daily | [1] |
| CHIRPS v2.0 | the Climate Hazards Group Infrared Precipitation with Stations, interpolated gauge observations combined with satellite data | 1981-2018 | 0.25° × 0.25° | daily | [2] |
| GPCC v2020 | the Global Precipitation Climatology Centre, interpolated gauge observations | 1982-1980 | 1.0° × 1.0° | daily | [3] |
| CPC-U | Climate Prediction Center Unified gauge-based analysis, interpolated gauge observations combined with satellite data | 1979-2018 | 0.50° × 0.50° | daily | [4] |
| ERA-5 | the fifth generation ECMWF global atmospheric reanalysis | 1979-2018 | 0.25° × 0.25° | hourly | [5] |
| PGF / GLDAS-v2.0^a^ | Princeton Global Forcings, by combining a suite of global observation-based datasets with the (NCEP–NCAR) reanalysis CRU, SRB/UMD, TRMM and NCEP/NCAR  NASA Global Land Data Assimilation System Version 2 (GLDAS-2), forced entirely with the Princeton meteorological forcing input data and provides a temporally consistent series from 1948 through 2014 | 1979-2016;  1979-2014 | 0.25° × 0.25° | daily | [6-7] |
| MERRA-2 | The Modern-Era Retrospective Analysis for Research and Applications, version 2, atmospheric reanalysis of the modern satellite era | 1980-2018 | 0.625° × 0.5° | daily | [8] |
| MSWEP v2.8 | Multi-Source Weighted-Ensemble Precipitation, optimally merging a range of gauge, satellite, and reanalysis estimates | 1979-2018 | 0.10° × 0.10° | 3-hourly | [9] |
| *Evapotranspiration* | | | | | |
| GLEAM v3.3a^*^ | the Global Land Evaporation Amsterdam Mode, based on reanalysis net radiation and air temperature, satellite and gauged-based variables | 1980-2018 | 0.25° × 0.25° | daily | [10] |

a, Because the PGF don’t have the net shortwave and longwave radiation output, we used the net radiation from land data assimilation systems GLDAS-v2.0 instead, which was forced entirely with the PGF input data.

**Supplementary Table 2. Regionally aggregated linear trends of DSL and Water Deficit.** The DSL and Water Deficit of four regions (southern Amazon, northern and southern central Africa, southwestern Africa) of each precipitation dataset are first spatially averaged, and their linear trends are assessed by the ordinary least squares linear regression for the period 1983-2016 (See Methods). Central Africa is divided into two parts, because the two dry seasons are different (northern and congo: DJF; southern and congo: JJA). The statistical significance level (*P* value) was determined by the two-tailed Student’s t-test and Mann–Kendall trend test. Significant (*P* < 0.05) changes are bold and insignificant changes are gray.

| **Regions** | **Datasets** | **DSL change (d decade^-1^)** | | | **WD change (mm decade^-1^)** | | |
| --- | --- | --- | --- | --- | --- | --- | --- |
|  |  | ***P < Ep*** | ***P < E*** | ***P < P*** | ***P < Ep*** | ***P < E*** | ***P < P*** |
| southern Amazonia (5°S-16°S; 48°W-65°W) | CHIRPS | 2.36 | 1.40 | 0.01 | **32.84** | 0.46 | 9.49 |
|  | GPCC | 3.63 | **3.75** | 1.05 | **39.93** | **6.80** | **18.40** |
|  | CPC-U | **10.29** | **11.19** | **8.69** | **57.10** | **21.96** | **40.09** |
|  | PGF | 3.58 | **3.32** | 0.17 | **38.32** | **6.06** | **12.44** |
|  | MERRA2 | **10.94** | **11.96** | **10.53** | **58.01** | **22.30** | **48.09** |
|  | MSWEP | 2.38 | 2.09 | 0.04 | **32.66** | 1.59 | 4.04 |
|  | PERSIANN | **4.81** | **3.65** | 3.30 | **38.27** | 3.93 | **19.32** |
|  | ERA5 | **8.82** | **10.71** | **5.95** | **64.98** | **26.10** | **43.03** |
| southern Africa  (23.5°S-13°S; 12°E-30°E) | CHIRPS | **-5.98** | **7.77** | -2.00 | 2.17 | **22.90** | 1.19 |
|  | GPCC | **-7.77** | **8.26** | -2.08 | -5.30 | **21.68** | 0.73 |
|  | CPC-U | -4.68 | **14.77** | 0.83 | 1.49 | **27.20** | 5.46 |
|  | PGF | **-7.93** | **5.30** | -4.45 | -7.56 | **20.57** | -4.56 |
|  | MERRA2 | -2.46 | **16.51** | 0.68 | 16.28 | **28.57** | 5.32 |
|  | MSWEP | **-9.24** | **7.23** | -3.58 | -10.97 | **19.15** | -1.20 |
|  | PERSIANN | **-6.07** | **9.14** | **-5.48** | -0.45 | **18.83** | 0.40 |
|  | ERA5 | **-5.66** | **15.88** | 1.85 | 15.66 | **27.23** | **9.04** |
| Congo Basin DJF dry season  (5°S-5°N; 10°E-30°E) | CHIRPS | 1.95 | -0.83 | -0.02 | 6.92 | -1.49 | -0.18 |
|  | GPCC | -0.52 | -2.84 | -1.62 | -2.96 | **-13.99** | -15.07 |
|  | CPC-U | **6.43** | 4.06 | **8.80** | **32.02** | **21.30** | **19.07** |
|  | PGF | 2.18 | -1.02 | -1.38 | 1.63 | -9.43 | -9.31 |
|  | MERRA2 | 5.21 | 3.35 | 4.54 | 18.52 | 9.40 | 15.20 |
|  | MSWEP | **13.19** | **10.75** | **13.39** | **34.79** | **21.37** | **33.17** |
|  | PERSIANN | 1.55 | -1.86 | -1.54 | 7.69 | -3.55 | -2.80 |
|  | ERA5 | **13.29** | **11.51** | **14.35** | **34.80** | **22.97** | **37.89** |
| southern Central African JJA dry season  (12°S-7°N; 15°E-30°E) | CHIRPS | 2.38 | 1.91 | 0.75 | **16.96** | **3.41** | -0.11 |
|  | GPCC | 1.98 | 1.12 | -0.16 | **18.91** | 4.85 | -3.10 |
|  | CPC-U | **10.89** | **11.65** | **10.69** | **48.88** | **37.40** | **23.99** |
|  | PGF | 1.61 | 0.85 | 0.68 | **12.82** | 2.24 | -5.35 |
|  | MERRA2 | **11.61** | **10.17** | **10.58** | **37.46** | **21.19** | **23.25** |
|  | MSWEP | **7.62** | **6.62** | **8.19** | **26.99** | **12.98** | **16.46** |
|  | PERSIANN | 2.17 | 1.79 | 1.33 | **19.76** | **7.50** | 4.39 |
|  | ERA5 | **10.20** | **11.01** | **11.93** | **38.43** | **23.05** | **30.97** |
| Sahel  (10°S-18°N; 18°W-20°E) | CHIRPS | **-4.19** | **2.96** | **-3.24** | -7.98 | **10.97** | **-3.45** |
|  | GPCC | **-3.99** | 0.75 | **-2.80** | -9.77 | **11.02** | -3.66 |
|  | CPC-U | -3.07 | **3.06** | -1.81 | -3.79 | **10.93** | -2.24 |
|  | PGF | -3.63 | -1.18 | **-4.00** | -7.41 | **8.29** | **-6.18** |
|  | MERRA2 | **-4.80** | **5.79** | -1.46 | -9.45 | **11.30** | -1.30 |
|  | MSWEP | -2.43 | **9.72** | 0.59 | 7.59 | **18.69** | 1.87 |
|  | PERSIANN | **-5.94** | 0.45 | **-3.88** | -13.70 | **8.07** | **-6.00** |
|  | ERA5 | 0.03 | **12.66** | **3.56** | 13.57 | **25.11** | **3.94** |

**
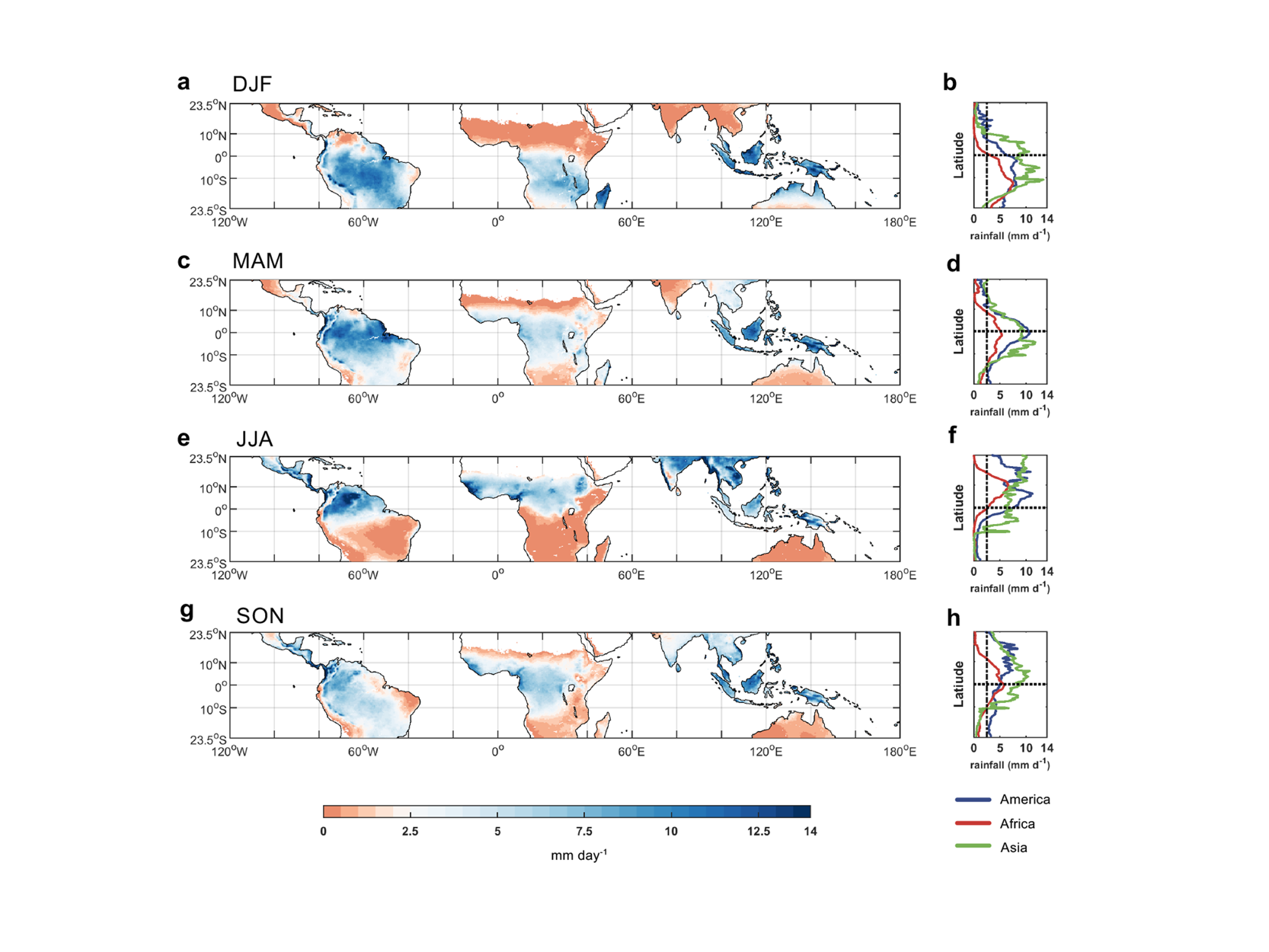
Supplementary Figure 1. Seasonal movement of tropical rain belt.** Mean rainfall rate during (**a-b)** December-January-February (DJF), (**c-d)** March-April-May (MAM), (**e-f)** June-July-August (JJA) and (**g-h)** September-October-November (SON), according to CHIRPS (1983-2016). In the figures on the right, the horizontal dashed line marks 0° latitude, and the vertical dashed line marks the rainfall rate of 2.5 mm day^-1^, which is usually used as the threshold to define the tropical rain belt^11^.


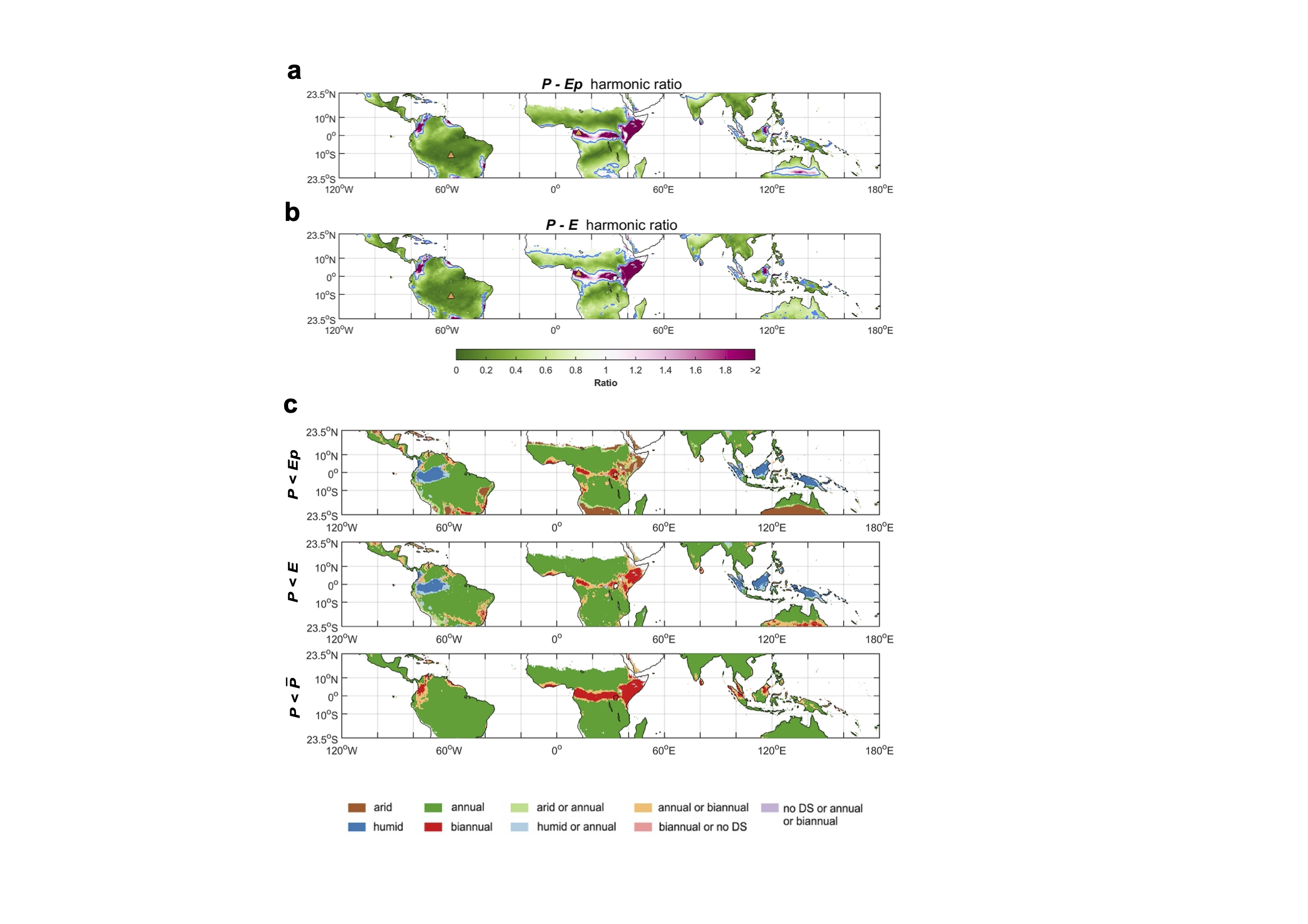
**Supplementary Figure 2. *P* < *Ep* and *P* < *E* occurrence (a, b) and number of dry seasons per year (c, d, e) across precipitation datasets. a-b,** The mean ratio of *P* - *Ep*, *P* - *E* amplitudes of the harmonics at frequencies of two and one cycles per year via Fourier Analysis of whole daily time series (1983-2016) for each grid box based on the seven precipitation datasets. The blue dashed line marks the boundaries of the area with ratio of 0.75.


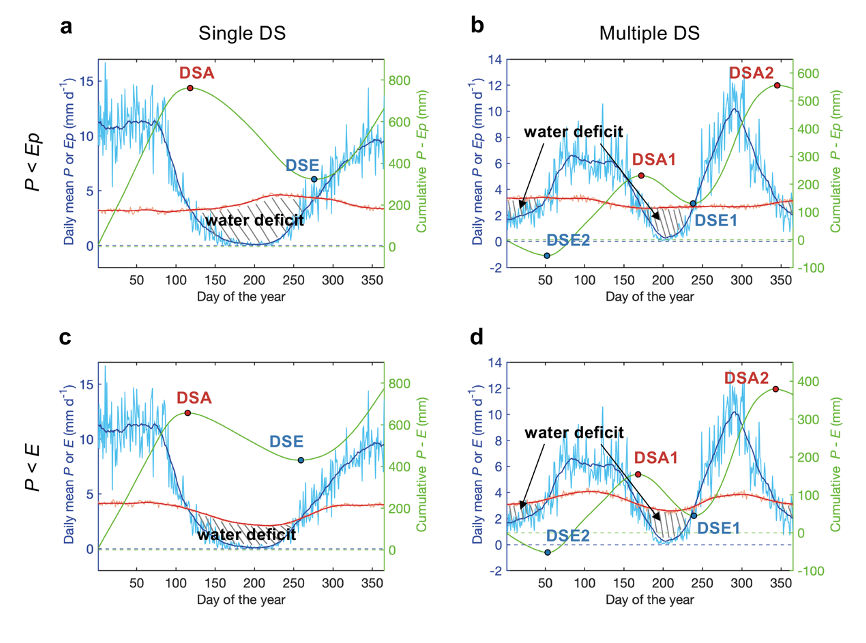
**Supplementary Figure 3. Dry seasons, defined as *P* < *Ep* (a, b) and *P* < *E* (c, d).** Daily mean rainfall (light blue) for each day of the year, smoothed using a 30-day running window (dark blue line), the climatological *Ep* or *E* (red line) and cumulative value (green line) for the grid box centered at 11.125°S, 57.875°W (left, point 1 in Fig. 1**a**) and 1.375°N, 12.875°E (right, point 2 in Fig. 1**a**) according to the daily CHIRPS dataset for the period 1983-2016 (see Eq. 1 and Eq. 2). Blue dots mark the arrival of dry seasons and red dots mark the end of dry seasons. The solid black shaded area represents the water deficit. The longest dry season and the longest wet season are assumed to be the seasons of interest for regions with multiple dry and wet seasons per year (**b, d**).


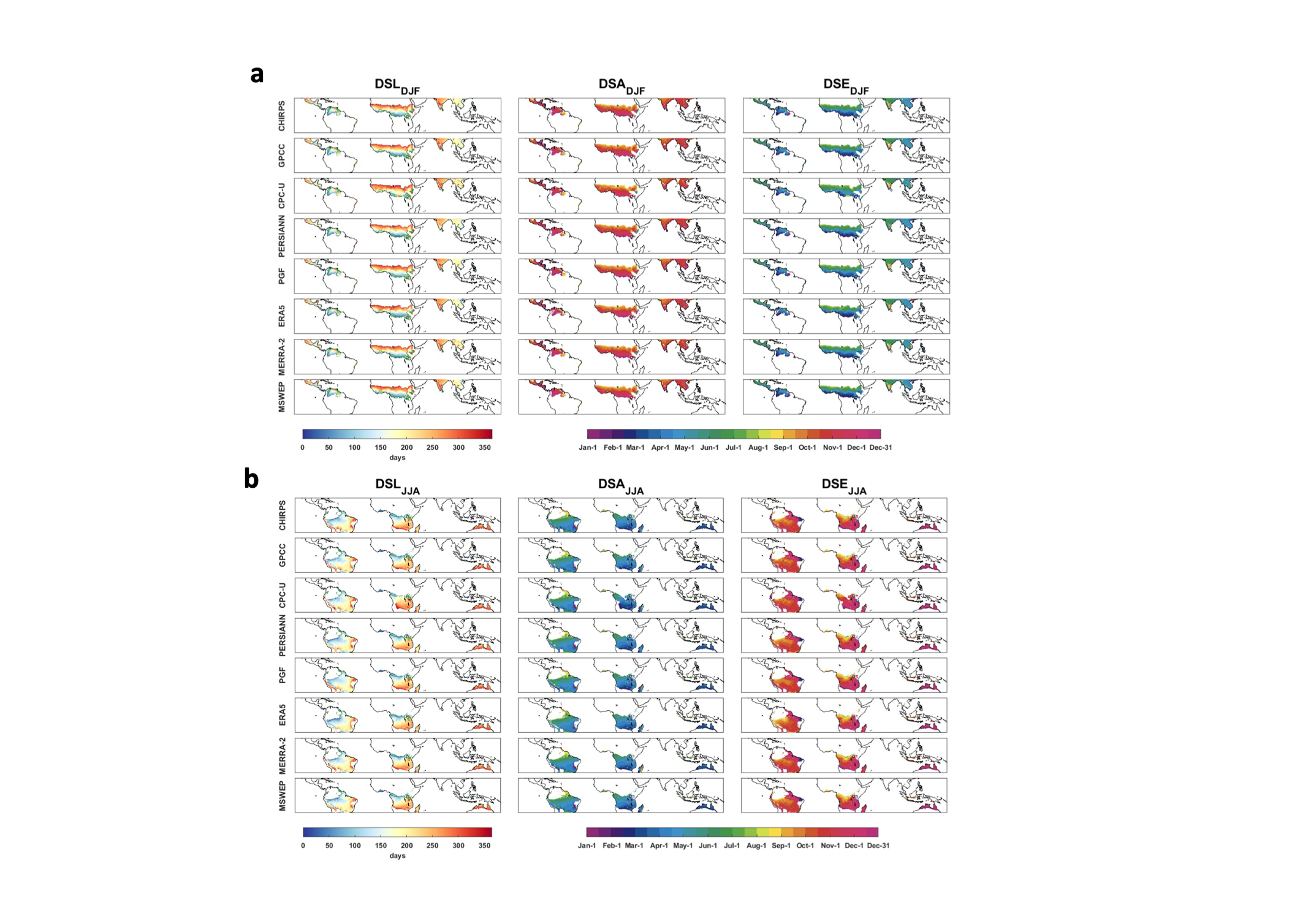

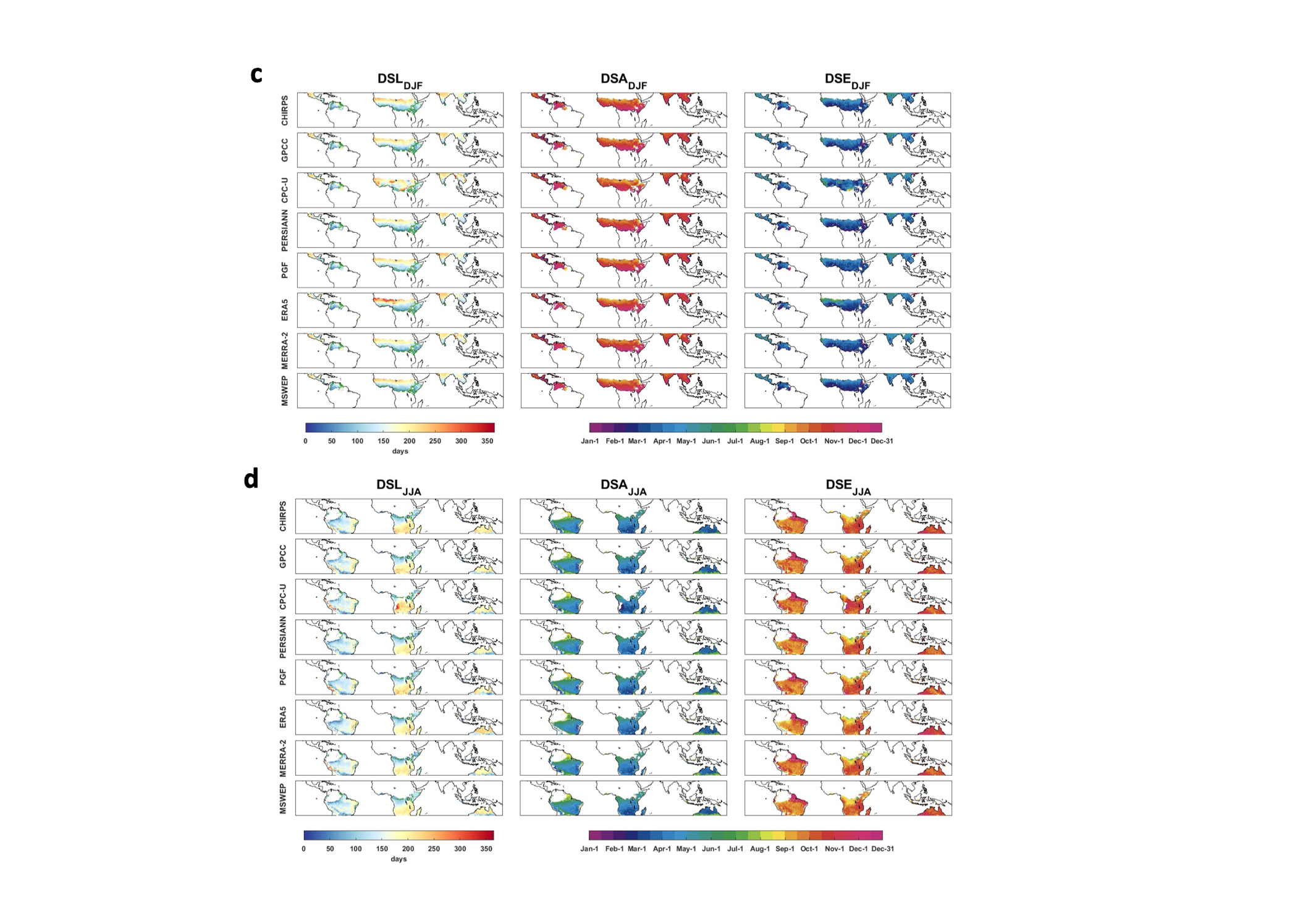


**
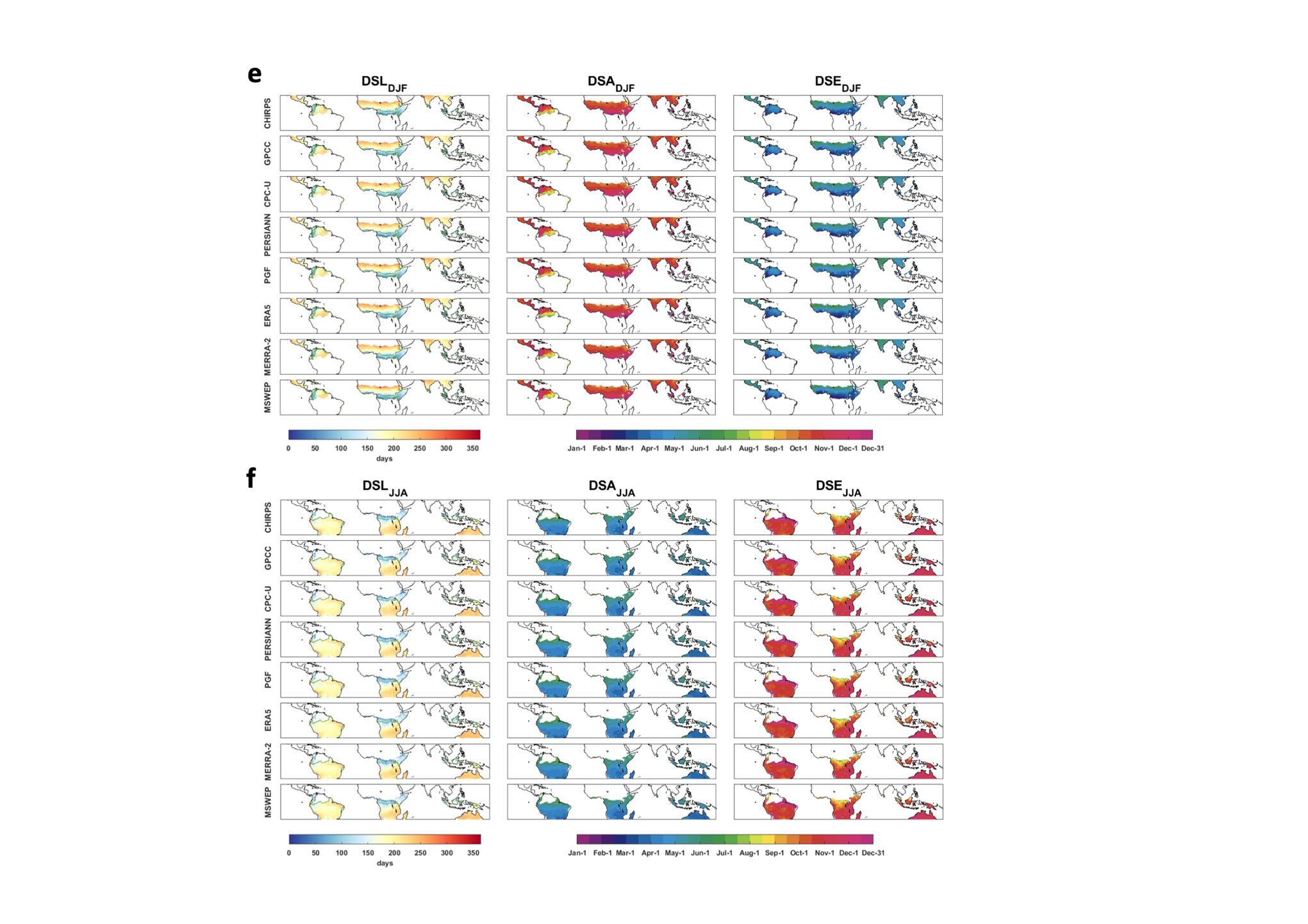

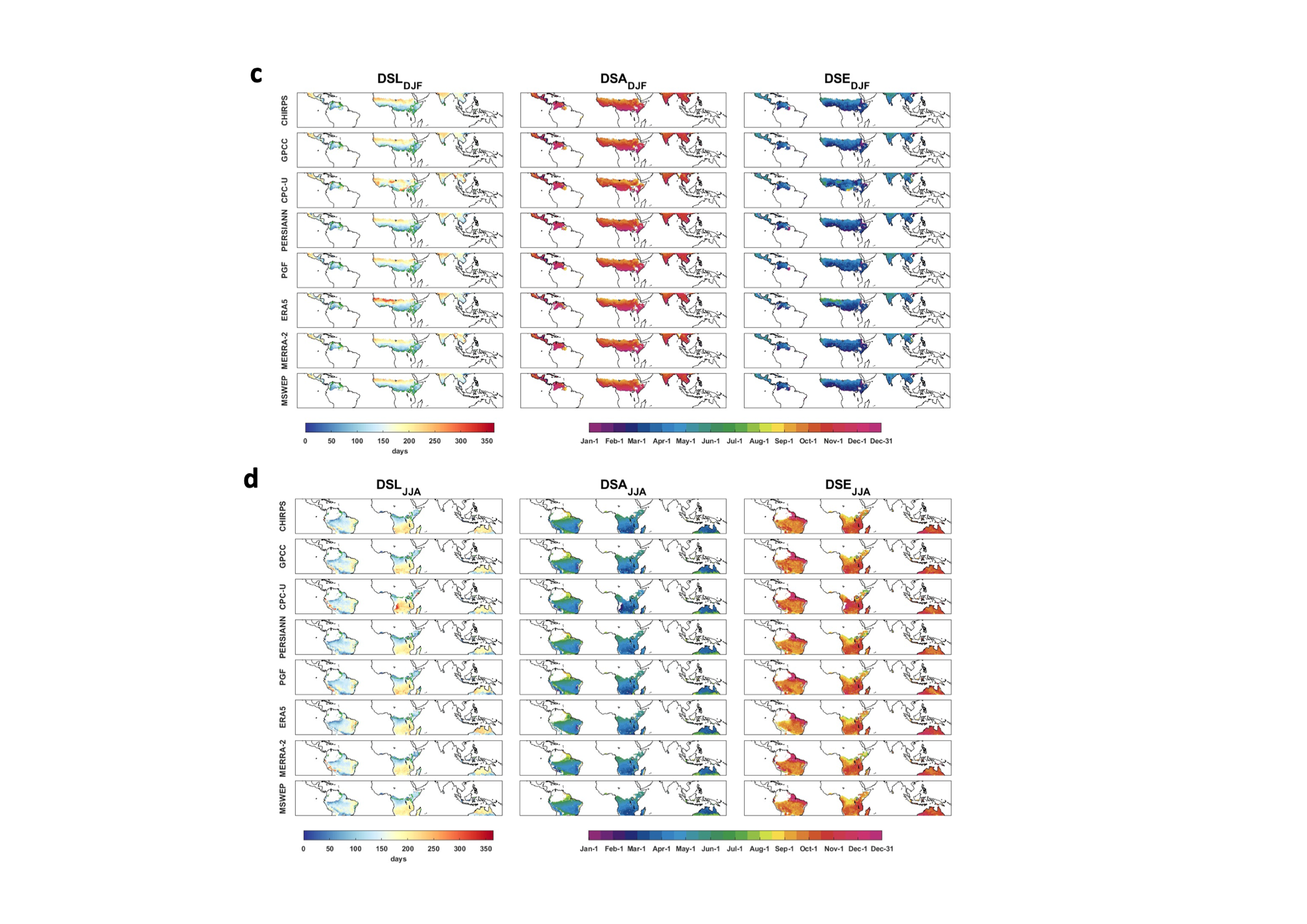
Supplementary Figure 4. Dry season length, arrival, and end, when defined as *P* < *Ep* (a-b), *P* < *E* (c-d) and *P* <** $\bar{\boldsymbol{P}}$ **(e-f), for each precipitation dataset across northern tropics.** The green line indicates the boundary of the region with two dry season per year.

**
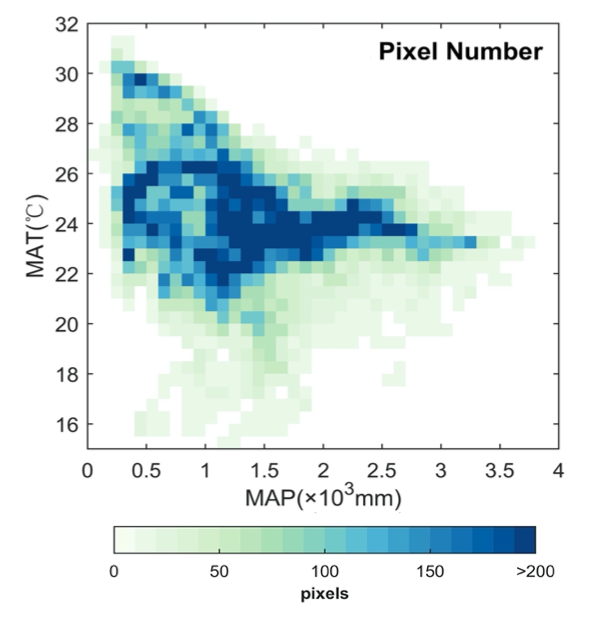
**

**Supplementary Figure 5**. **Pixels distribution for each MAT and MAP interval shown in Figure 3a.**


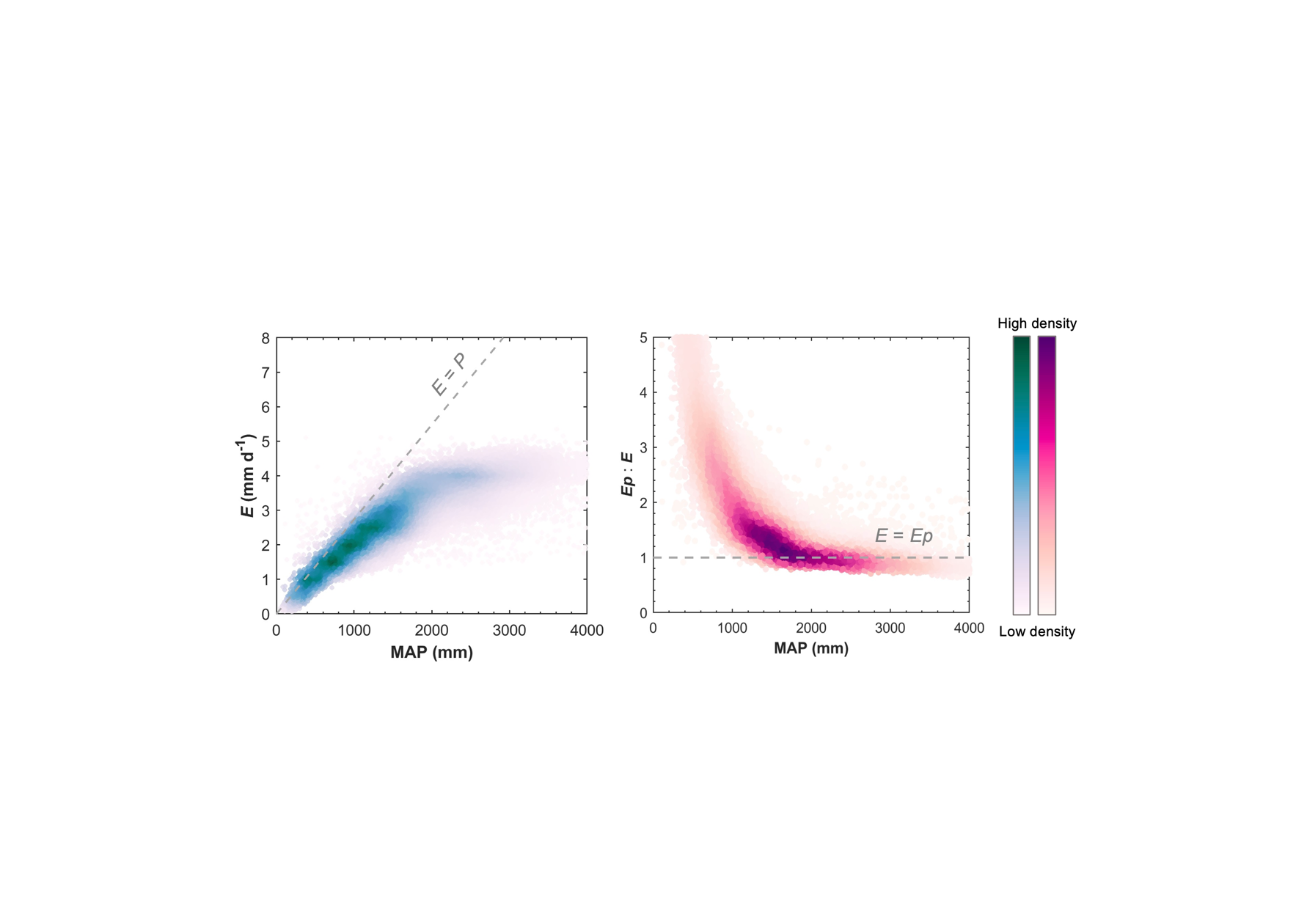
**Supplementary Figure 6**. **Complementary relationship between *Ep*, *E* and multi-year average.** Point cloud of climatological *Ep*, *E* and MAP during 1983-2016 for all grids in tropics. *Ep*, *E* and MAP were derived from ERA5, GLEAM and MSWEP, respectively. Gray dashed lines show the identity line.

**
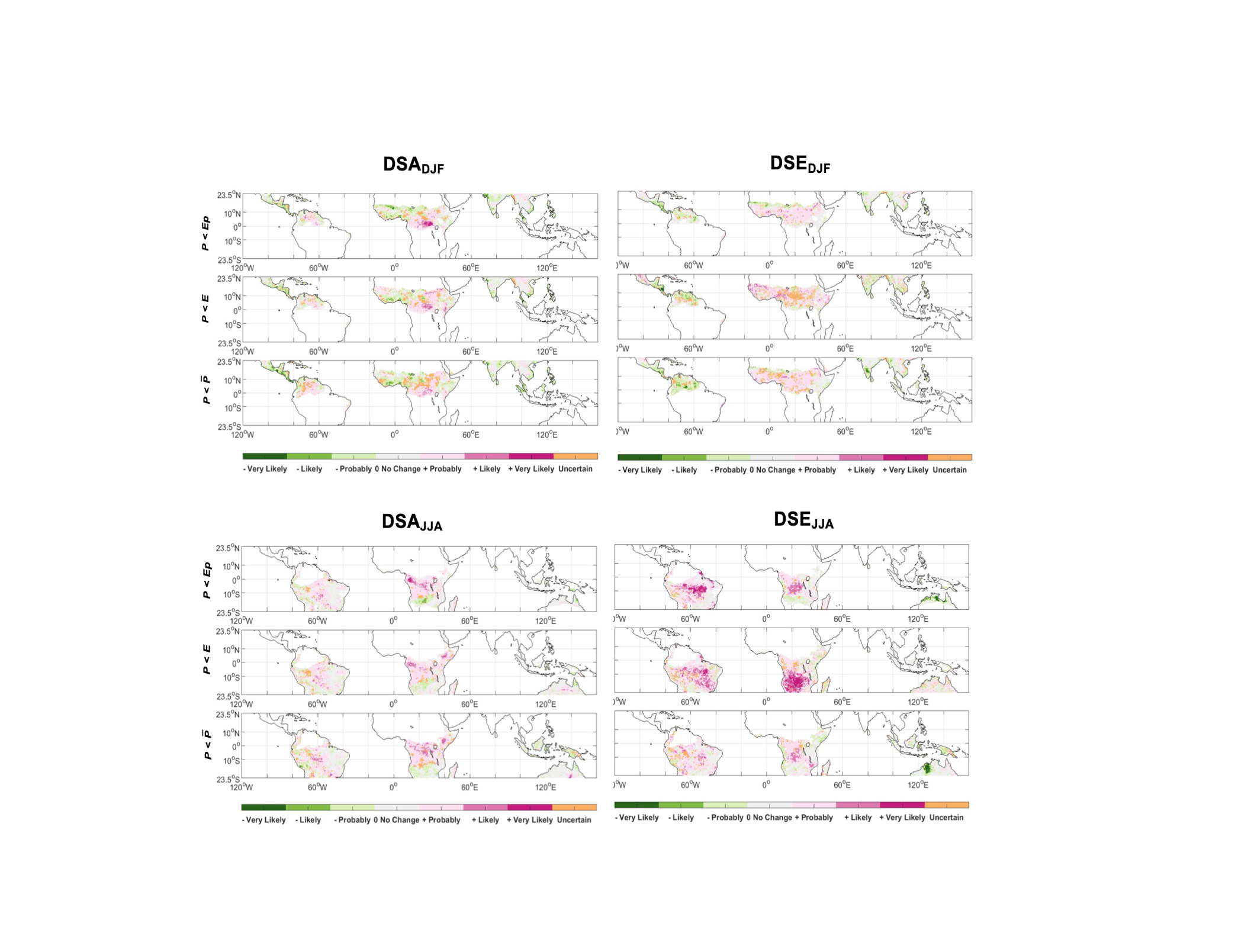
Supplementary Figure 7**. **Consistency of linear trends in DSA and DSE, among eight datasets and under three different definitions (1983-2016).** The consistency of wetting (green) or drying (pink) trends under each of the three definitions of “dry season” was assessed as the variation among the eight datasets. ‘‘Very likely’’, ‘Likely’’ and ‘‘Probably” indicate that the sign of the trend was the same and significant in six - eight, four - five and one to three precipitation datasets, respectively, while the other datasets showed no significant change. “Uncertain’’ indicates conflicting trends among datasets, with some showing a significant increase and some showing a significant decrease. “No Change” indicates that all eight datasets showed no significant change.

**
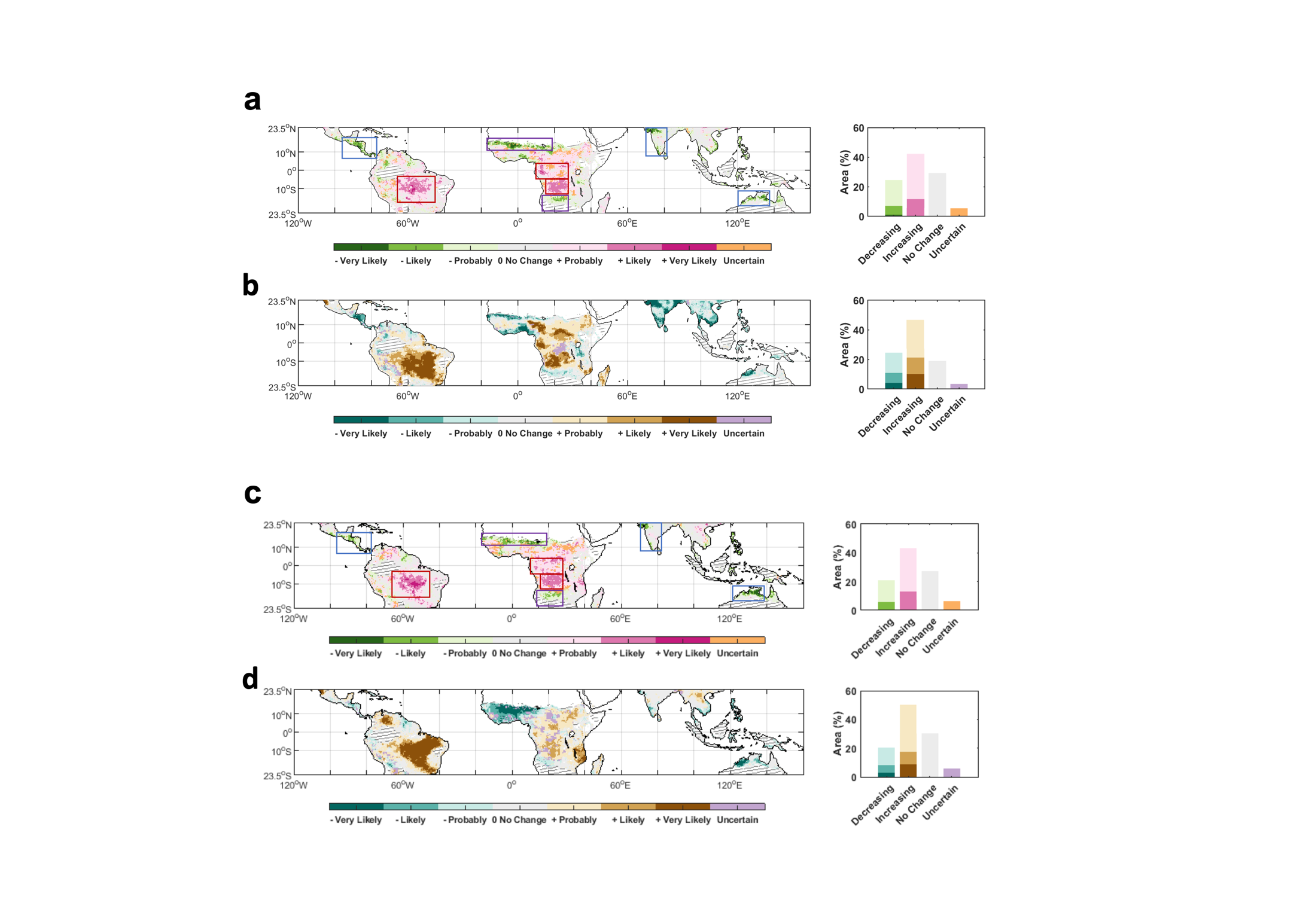
Supplementary Figure 8. Trends in DSL(a, c), WD (b, d) under the definitions *P* < *Ep*  for other two *Ep* datasets.** Results based on the *Ep* calculated from MERRA-2 was shown in **a-b**, and results based on the *Ep* calculated from PGF and GLDAS v2.0 wad shown in **c-d.**

**
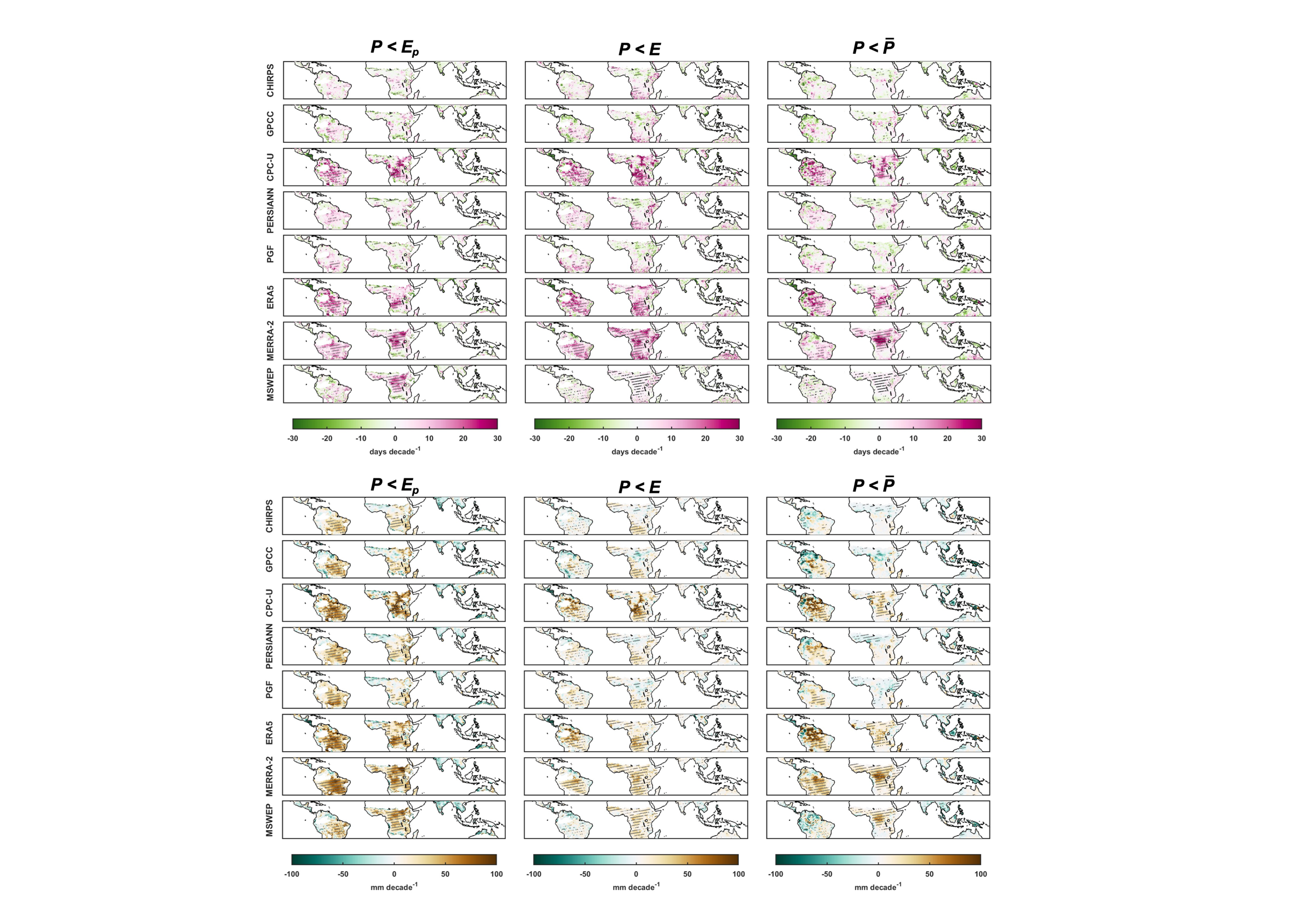
Supplementary Figure 9. Trends in DSL, DSA, DSE (a-c) under the definitions *P* < *Ep, P* < *E, P* <** $\bar{\boldsymbol{P}}$***,* respectively*,* and Water Deficit (d), under each definition, and for each precipitation dataset.** The hatched area in each pattern indicates where the trend is significant (*P* < 0.05).

| **Model name** | **Model name** |
| --- | --- |
| ACCESS-CM2 | FGOALS-g3 |
| ACCESS-ESM1-5 | GFDL-CM4 |
| AWI-ESM-1-1-LR | HadGEM3-GC31-LL |
| BCC-CSM2-MR | HadGEM3-GC31-MM |
| CESM2 | INM-CM4-8 |
| CESM2-FV2 | INM-CM5-0 |
| CESM2-WACCM | KACE-1-0-G |
| CESM2-WACCM-FV2 | MIROC-ES2L |
| CMCC-CM2-SR5 | MPI-ESM-1-2-HAM |
| CNRM-CM6-1 | MPI-ESM1-2-HR |
| CNRM-CM6-1-HR | MPI-ESM1-2-LR |
| CNRM-ESM2-1 | MRI-ESM2-0 |
| CanESM5 | NorESM2-LM |
| EC-Earth3 | NorESM2-MM |
| EC-Earth3-Veg | SAM0-UNICON |
| EC-Earth3-Veg-LR | TaiESM1 |
| FGOALS-f3-L | UKESM1-0-LL |

**Supplementary Table 3. List of CMIP6 models used in this study**.


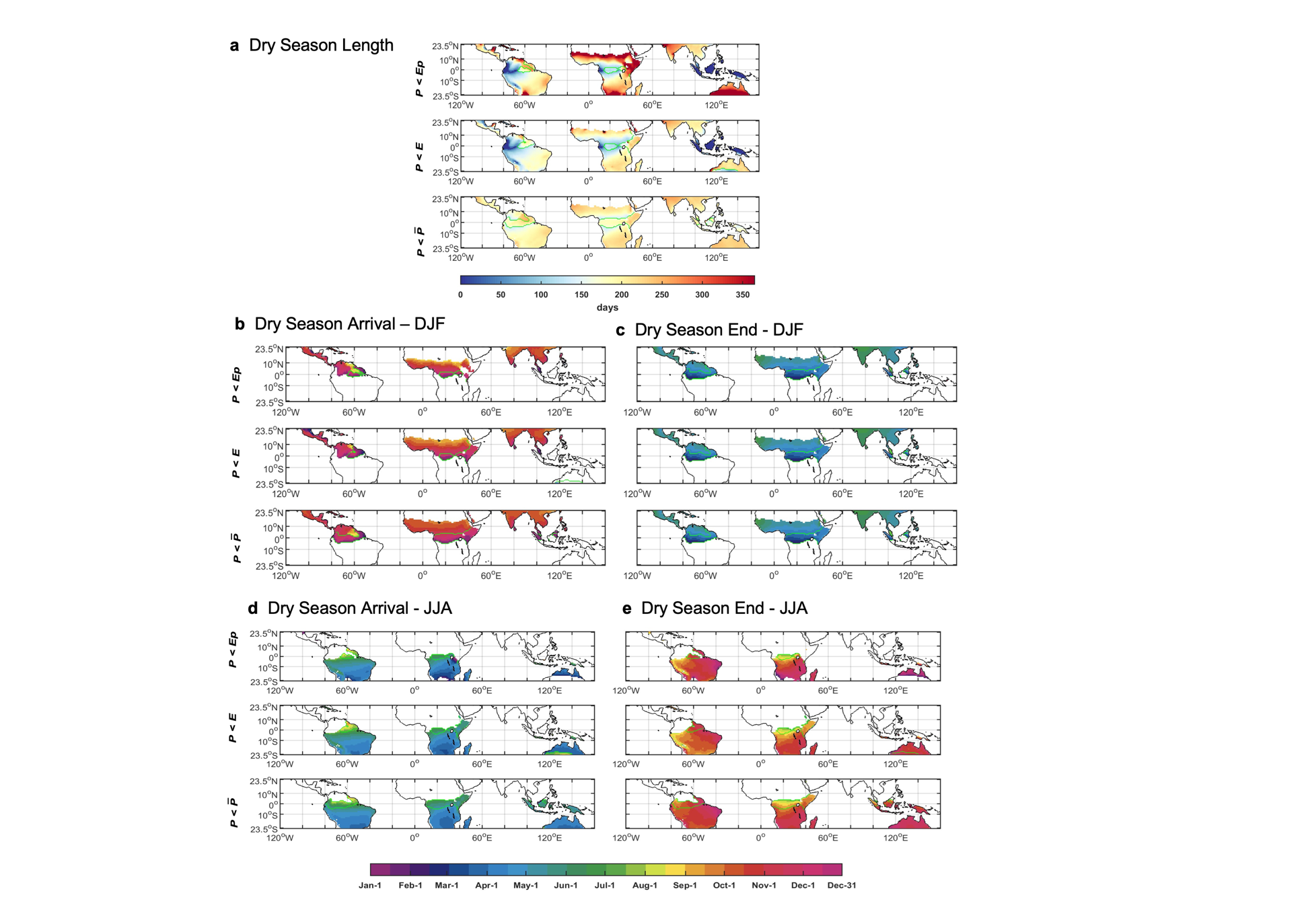
**Supplementary Figure 10. Spatial pattern of multi-model mean dry season length and timing in tropics under three definitions.** The model results are from historical simulation (1983-2014) of 34 CMIP6 climate models^12^. The green contour line shows the boundary of regions with two dry seasons annually, usually with a boreal winter (December – February) dry season in **b-c** and a boreal summer (June – August) dry season **d-e**.

**
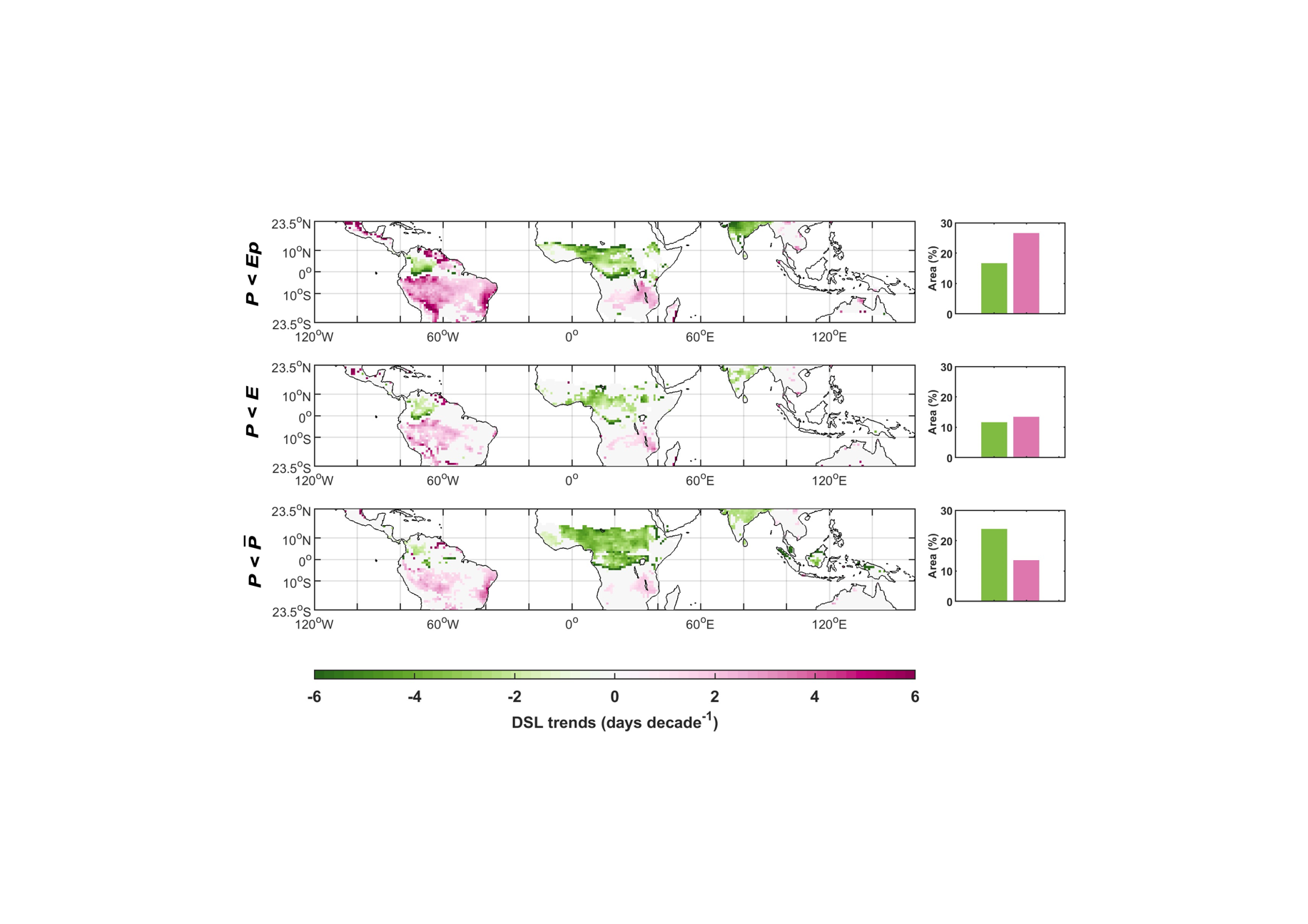
**

**Supplementary Figure 11**. **Multi-model mean trends in DSL for each definition**. The model results are from historical simulation (1983-2014) of 34 CMIP6 climate models. Only linear trends that are statistically significant at *P* *<* 0.05 are shown. The histogram shows the percent area with significant wetting or drying trends.

**
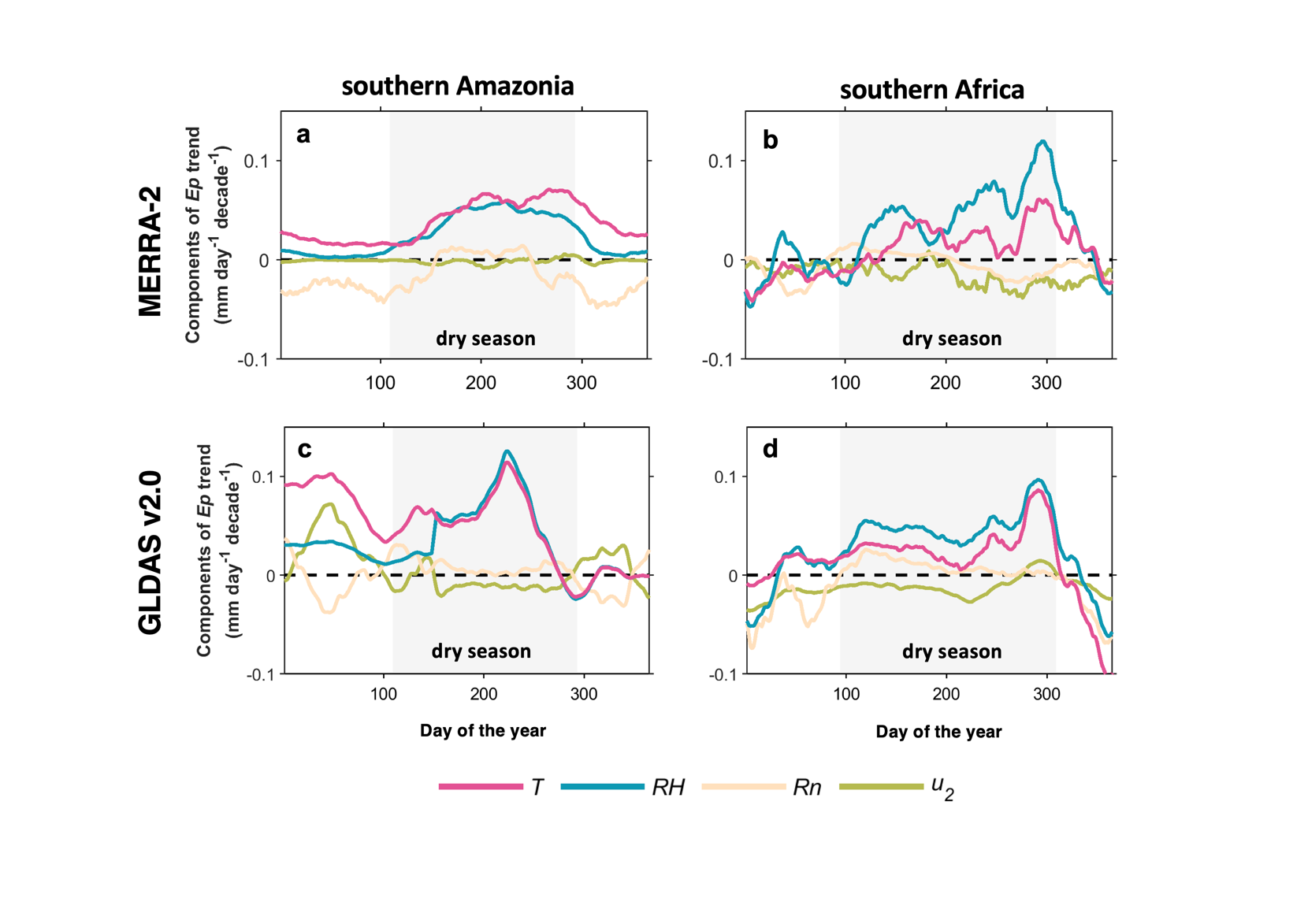
Supplementary Figure 12. The drivers of seasonal trajectories of *Ep* changes a in southern Amazonia and southern Africa for MERRA-2 and GLDASv2.0 datasets.** For each day of the year, the linear trends were calculated with the zonally averaged and 30-day smoothed daily values over southern Amazonia (48-65°W, 5-16°S, **a, c**) and southern Africa (12-30°E, 13-23.5°S, **b, d**), over the 1983-2016 period. The gray area indicated the dry season based on *P* < $\bar{P}$. Panels **a-d** show the individual contributions of *T*, *RH*, *u_2_* and *Rn* to the overall trend of *Ep*.


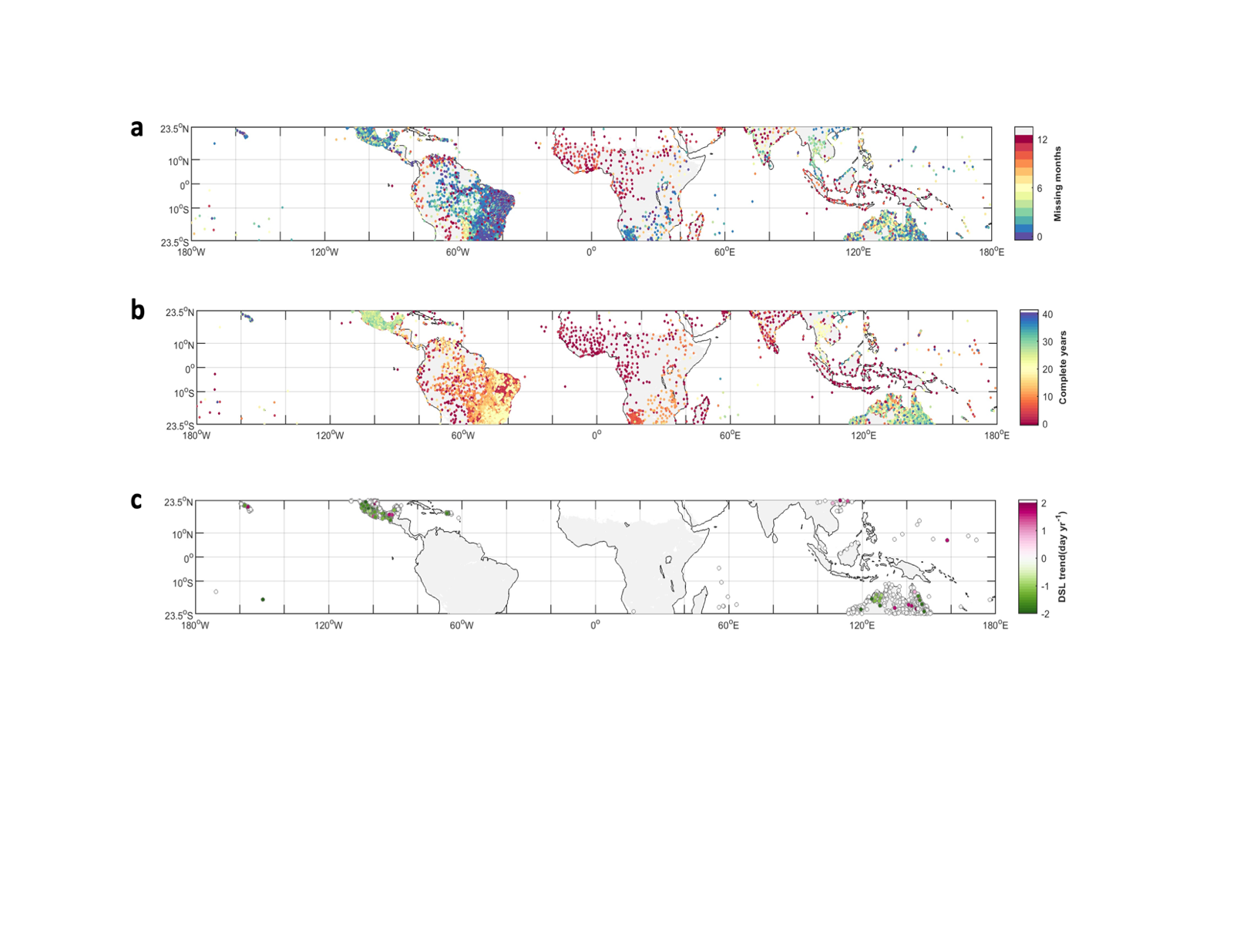
 **Supplementary Figure 13. Data quality of meteorological stations in GHCN-Daily and its *P*-based dry season length trend. a,** average missing month per year during their recorded years. **b,** complete year^13,14^ number during 1983-2018 for each station. **c,** *P*-based DSL trend for stations with at least 25 complete years during 1983-2018.

**Supplementary Reference**

1. Ashouri, H., et al. PERSIANN-CDR Daily Precipitation Climate Data Record from Multisatellite Observations for Hydrological and Climate Studies. *Bull. Am. Meteorol. Soc.* **96**, 69-83 (2015).

2. Funk, C. et al. The climate hazards infrared precipitation with stations-a new environmental record for monitoring extremes. *Sci. Data* **2**, 150066 (2015).

3. Ziese, M et al. *GPCC Full Data Daily Version.2018 at 1.0°: Daily Land-Surface Precipitation from Rain-Gauges built on GTS-based and Historic Data.* (GPCC, 2018) <http://dx.doi.org/10.5676/DWD_GPCC/FD_D_V2018_100>

4. Chen, M. Y. et al. Assessing objective techniques for gauge-based analyses of global daily precipitation. *J. Geophys. Res. Atmos.* **113**, D04110 (2008).

5. Dee, D. P. et al. The ERA-Interim reanalysis: configuration and performance of the data assimilation system. *Q. J. R. Meteorolog. Soc.* **137**, 553-597 (2011).

6. Sheffield, J., Goteti, G. & Wood, E. F. Development of a 50-year high-resolution global dataset of meteorological forcings for land surface modeling. *J. Clim.* **19**, 3088-3111 (2006).

7. Rodell, M., et al. The global land data assimilation system. *Bull. Am. Meteorol. Soc.* **85**, 381-394 (2004).

8. Gelaro, R. et al. The Modern-Era Retrospective Analysis for Research and Applications, Version 2 (MERRA-2). *J. Clim.* **30**, 5419-5454 (2017).

9. Beck, H. E., et al. MSWEP V2 Global 3-Hourly 0.1° Precipitation: Methodology and Quantitative Assessment. *Bull. Am. Meteorol. Soc.* **100**, 473-500 (2019).

10. Martens, B. et al. GLEAM v3: satellite-based land evaporation and root-zone soil moisture. *Geosci. Model Dev.* **10**, 1903-1925 (2017).

11. Jiang, Y. et al. Widespread increase of boreal summer dry season length over the Congo rainforest. *Nat. Clim. Change* **9**, 617-622 (2019).

12. Eyring, V., et al. Overview of the Coupled Model Intercomparison Project Phase 6 (CMIP6) experimental design and organization. *Geosci. Model Dev.* **9**, 1937-1958 (2016).

13. Menne, M. J., Durre, I., Vose, R. S., Gleason, B. E., Houston, T. G. An Overview of the Global Historical Climatology Network-Daily Database. *J. Atmos. Oceanic Technol.* **29**, 897-910 (2012).

14. Contractor, S., et al. Rainfall Estimates on a Gridded Network (REGEN) – a global land-based gridded dataset of daily precipitation from 1950 to 2016. *Hydrol. Earth Syst. Sci.* **24**, 919-943 (2020).
